# Identification of the amino acid position controlling the different enzymatic activities in walnut tyrosinase isoenzymes (*jr*PPO1 and *jr*PPO2)

**DOI:** 10.1038/s41598-020-67415-6

**Published:** 2020-07-02

**Authors:** Felix Panis, Annette Rompel

**Affiliations:** Universität Wien, Fakultät für Chemie, Institut für Biophysikalische Chemie, Althanstraße 14, 1090 Wien, Austria

**Keywords:** Biochemistry, Enzymes, Oxidoreductases, Chemistry, Chemical biology, Enzymes, Oxidoreductases

## Abstract

Polyphenol oxidases (PPOs) are ubiquitously distributed among plants, bacteria, fungi and animals. They catalyze the hydroxylation of monophenols (monophenolase activity) and the oxidation of *o*-diphenols (diphenolase activity) to *o*-quinones. PPOs are commonly present as an isoenzyme family. In walnut (*Juglans regia*), two different genes (*jr*PPO1 and *jr*PPO2) encoding PPOs have been identified. In this study, *jr*PPO2 was, for the first time, heterologously expressed in *E. coli* and characterized as a tyrosinase (TYR) by substrate scope assays and kinetic investigations, as it accepted tyramine and L-tyrosine as substrates. Moreover, the substrate acceptance and kinetic parameters (*k*_cat_ and *K*_m_ values) towards 16 substrates naturally present in walnut were assessed for *jr*PPO2 (TYR) and its isoenzyme *jr*PPO1 (TYR). The two isoenzymes prefer different substrates, as *jr*PPO1 shows a higher activity towards monophenols, whereas *jr*PPO2 is more active towards *o*-diphenols. Molecular docking studies performed herein revealed that the amino acid residue in the position of the 1st activity controller (His_B1_ + 1; in *jr*PPO1 Asn240 and *jr*PPO2 Gly240) is responsible for the different enzymatic activities. Additionally, interchanging the 1st activity controller residue of the two enzymes in two mutants (*jr*PPO1-Asn240Gly and *jr*PPO2-Gly240Asn) proved that the amino acid residue located in this position allows plants to selectively target or dismiss substrates naturally present in walnut.

## Introduction

**Figure 1:**
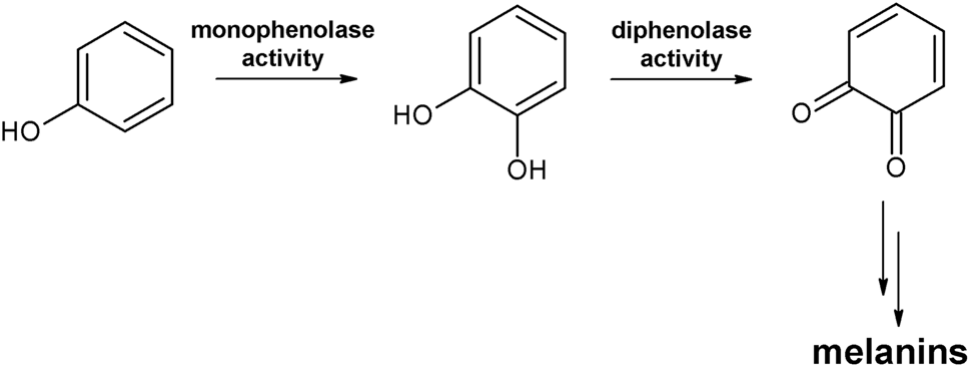
Reactions catalyzed by PPOs. COs catalyze solely the oxidation of *o*-diphenols to the respective *o*-quinones (diphenolase activity, catecholase activity), whereas TYRs catalyze the *o*-hydroxylation of monophenols (monophenolase activity, cresolase activity) as well as the subsequent two-electron oxidation of the resulting *o*-diphenols to the respective *o*-quinones, which non-enzymatically form melanins.

Polyphenol oxidases (PPOs) are metalloenzymes with a type-III copper center widely distributed among archaea, bacteria, fungi, animals and plants^1–3^. PPOs consist of tyrosinases (TYRs), catechol oxidases (COs) and aurone synthase (AUS). TYRs catalyze the *ortho*-hydroxylation of monophenols to *o*-diphenols (monophenolase activity, EC 1.14.18.1) as well as their subsequent oxidation to *o*-quinones (diphenolase activity, EC 1.10.3.1), whereas COs are solely able to perform diphenolase activity (Fig. 1). AUS is involved in secondary plant metabolism by producing aurones^1,4^. *O*-quinones are highly reactive and spontaneously polymerize, leading to the formation of melanins, which in plant products are associated with a reduced concentration of bioactive compounds (phenols, flavonoids, condensed tannins)^5^ and a reduced economic value^6^.

Walnut tyrosinase (*jr*PPO1) has been studied extensively^7–12^. In vivo, *jr*PPO1 is expressed as a latent 66.8 kDa pre-pro-enzyme composed of the catalytically active domain (~ 39 kDa) which is flanked by an N-terminal chloroplast transit peptide (~ 12 kDa) and a C-terminal domain (~ 16 kDa) that is shielding the entrance to the catalytic pocket and requires removal for the enzymatic activity to occur^7,8^. As shown for apple tyrosinase (*Md*PPO1), this can be achieved by a self-cleavage reaction^13^. Alternatively, PPOs can be activated in vitro by fatty acids^14^, acidic pH^15^, proteases^16,17^ and detergents such as sodium dodecyl sulfate (SDS)^10,18^. The crystal structure of *jr*PPO1 unveiled the architecture of the di-copper center in which each of the two copper atoms (CuA and CuB) is coordinated by three conserved histidine residues^9^. Comparison of the active centers of *jr*PPO1 and other plant PPOs shows a high level of conservation, however, two amino acid residues, named 1st (His_B1_ + 1 = His239 + 1) and 2nd (His_B2_ + 1 = His243 + 1) activity controller residue (Fig. S1), which are located in proximity to the di-copper center, are less conserved among plant PPOs and have been shown recently to control mono- and diphenolase activity in *jr*PPO1^10^.

In 2016 Martínez-García et al.^19^ identified a second tyrosinase gene within the walnut genome, encoding a putative TYR (*jr*PPO2), which is, for the first time, investigated within this study.

*jr*PPO1 (C0LU17) and *jr*PPO2 (A0A2I4DDQ0) share an amino acid sequence identity of 73% (Fig. S2) and especially the active centers show a high level of conservation (Fig. S3). The most striking difference between the two active sites is the amino acid in the position of the 1st activity controller: *jr*PPO1 features an asparagine (Asn240), whereas *jr*PPO2 displays a glycine (Gly240). Asn in the position of the 1st activity controller (His_B1_ + 1) has previously been associated with increased monophenolase activity as it stabilizes a water molecule which is activated by a conserved glutamic acid and reacts as a base for the deprotonation of incoming substrates. The deprotonation of the substrate is imperative for the hydroxylation of monophenols^20^. Thus, an asparagine in the position of the 1st activity controller is also present in the sequences of *Md*PPO2^21^ (*Malus domestica;* TYR) and *Vv*PPOg^22^ (*Vitis vinifera,* TYR). In contrast, the deprotonation of a substrate can also occur by a hitherto unknown mechanism as glycine in the position of the 1st activity controller is compatible with monophenolase activity as well. This has already been proven by the sequences of *Md*PPO3^21^ (*Malus domestica;* TYR), *To*PPO1^23^, *To*PPO2^23,24^ (*Taraxacum officinale,* TYR) and *Vv*PPOcs-3 (*Vitis vinifera,* TYR)^25^, which all accept the standard substrates L-tyrosine and/or tyramine.

Walnut (*Juglans regia*) is known for being rich in phenolic compounds valuable to the cosmetic and pharmaceutical industries^5,26^, however, PPO side reactions in walnut limit the availability of these compounds^27,28^. The rich abundance of phenolic compounds in walnut has been investigated thoroughly within the last years^29–33^, leading to the identification of numerous small phenolic compounds (e.g. pyrogallol, benzoic acid derivatives, phenylacetic acid derivatives and cinnamic acid derivatives; Fig. S4). Besides, flavonoids represent a prominent group of phenolics in walnut (Fig. S5), represented by the flavonols kaempferol, quercetin and myricetin and the flavanonol taxifolin, which are associated with positive health effects, such as antioxidative, antibacterial, antitumoral and anti-inflammatory activity^34–37^. Thus, their preservation is desired upon storage. Moreover, naphthoquinones, with juglone (5-hydroxy-1,4-naphthoquinone; Fig. S5) being the most abundant one, are phenolic compounds characteristic for *Juglans regia*^29^. They have been demonstrated to show allelopathic, insecticidal and anthelmintic effects^38–40^ and are, therefore, proposed as biological insecticides and herbicides^39^. Moreover, the cultivation of walnut has gained economic relevance within the last decades due to timber production as well as the high nutritional value of walnut kernels^26,30,41^. However, little is known about the tyrosinase activity towards the vast spectrum of phenolic compounds naturally present in walnut. Thus, understanding the reactivities of *jr*PPO1 and *jr*PPO2 offers the possibility of controlling the PPO activity in walnut.

Herein, we report the cloning of the gene encoding latent *jr*PPO2, the recombinant expression of soluble protein as well as its biochemical characterization. Moreover, the activity of recombinantly expressed *jr*PPO1^10^ and *jr*PPO2 towards natural walnut substrates was assessed and the activities of the two enzymes clearly showed different substrate preferences. Kinetic investigations supplemented with docking studies identified the 1st activity controller residue (*jr*PPO1: Asn240, *jr*PPO2: Gly240) as the cause for the different reactivities in these two enzymes, which was further substantiated by kinetic measurements and docking studies using two mutants targeting the 1st activity controller residues of the two isozymes (*jr*PPO1-Asn240Gly, *jr*PPO2-Gly240Asn).

## Results and discussion

### Genomic DNA extraction and cloning of the *jr*PPO2 gene

Genomic DNA (gDNA) was isolated from walnut leaves using a cetyltrimethylammonium bromide (CTAB) assisted cell lyses method, which produced a total yield of 250 ng gDNA (~ 40.000 base pairs)/g frozen plant material. The co-extraction of phenolic compounds substantially decreases downstream applicability (PCR) of DNA extracts. Thus, 2% (w/v) PVP (polyvinylpyrrolidone) was added to the DNA extraction buffer^42^ as well as 20 mM sodium ascorbate to suppress PPO side reactions since in situ produced quinones oxidize DNA.

The predicted *jr*PPO2 gene^19^ encompasses an N-terminal chloroplast transit peptide, an active domain and a C-terminal domain. Using *Q5 High-Fidelity DNA polymerase* and specific primers (Table S1) a ~ 1,700 base pair amplicon was obtained, cloned in the pENTRY-IBA51 vector and sequenced to reveal the sequence of the predicted *jr*PPO2. Compared to the sequence published by Martinez-Garcia et al.^19^, the gene sequenced herein contained the following mutations: Asp256Asn, Phe293Leu, Ser296Pro and Asp477Asn, which are all located on the surface of the protein and are a result of different habitats of sampled trees (Vienna, Austria (this study) vs. California^19^). Minor structural variability has already been reported for *jr*PPO1, however, enzymatic activity, pH optimum and SDS dependent activation of the latent enzyme has been shown to remain unaffected by these variations^10^.

Based on the sequencing results, a second primer pair was designed binding to the starting region of the active domain and the end of the C-terminal domain (Table S1), which produced an amplicon corresponding to full-length *jr*PPO2 (active domain and C-terminal domain; Fig. S2). After cloning into a pGEX vector the construct was transformed into competent *E. coli* BL21 (DE3) cells and used for protein expression. The enzyme was expressed as a fusion protein with an N-terminal GST-tag, which on the one hand facilitates its purification and on the other hand increases the solubility and, thus, reduces the formation of inclusion bodies^21,43^.

### Expression of *jr*PPO1, *jr*PPO1-Asn240Gly, *jr*PPO2 and *jr*PPO2-Gly240Asn

As described previously for *jr*PPO1^10^ and other plant PPOs^21,44^, expression at low temperatures (~ 20 °C) in combination with prolonged expression times and the usage of a nutrient-rich medium (2xYT) results in an increased overall yield. *jr*PPO2 produced the highest yield with 70 mg/l purified, latent enzyme, followed by *jr*PPO2-Gly240Asn (63 mg/l), *jr*PPO1 (41 mg/l) and *jr*PPO1-Asn240Gly (34 mg/l). All enzymes were expressed at a purity level of at least 95% (Fig. S6) and were stored in 50 mM Tris–HCl pH 7.5 and 200 mM NaCl and were immediately used for kinetic measurements.

### Molecular mass determination

ESI-LTQ-MS revealed the masses of recombinant *jr*PPO1, *jr*PPO1-Asn240Gly, *jr*PPO2 and *jr*PPO2-Gly240Asn. The crystal structure analysis of *jr*PPO1^9^ exhibits one thioether bridge and two conserved disulfide bonds, which are, due to the similar spatial arrangement of the amino acids involved in the formation of the disulfide bonds and the thioether bridge, most probably also in vivo present in *jr*PPO2 (Fig. S1). The masses of *jr*PPO1, *jr*PPO2 and *jr*PPO2-Gly240Asn matched with the calculated masses corresponding to the formation of the thioether bridge and one of the two disulfide bonds being closed. The mass of *jr*PPO1-Asn240Gly indicated the formation of the thioether bridge and both disulfide bonds (Table 1 and Fig. S7) being closed. Varying numbers of closed disulfide bonds due to ESI–MS investigations have already been reported for *jr*PPO1^10^. The formation of the disulfide bonds during the recombinant expression process in *E. coli* is impeded by the reducing environment of the cytosol^45^. However, disulfide bonds can be present as an artifact of the electrospray ionization process, during which thiyl radicals, formed via one-electron oxidation of thiol groups, dimerize rapidly^46^. The thioether bridge is formed independently in the bacterial cytosol via an autocatalytic process after copper incorporation into the active center^47^. Thus, the disulfide bonds can be attributed to the ionization process whereas the thioether bridge is formed during the expression process.

**Table 1:** Calculated and measured molecular weights of *jr*PPO1, *jr*PPO1-Asn240Gly, *jr*PPO2 and *jr*PPO2-Gly240Asn.

Enzyme	Mass calculated (Da)	Mass measured (Da)	Δ_mass_ (Da)
*jr*PPO1	56,359.37 (–4H)	56,358.81	− 0.56
*jr*PPO1-Asn240Gly	56,300.31 (–6H)	56,300.58	+ 0.27
*jr*PPO2	56,790.82 (–4H)	56,790.56	− 0.26
*jr*PPO2-Gly240Asn	56,847.87 (–4H)	56,847.58	− 0.29

### Characterization of *jr*PPO2

**Figure 2:**
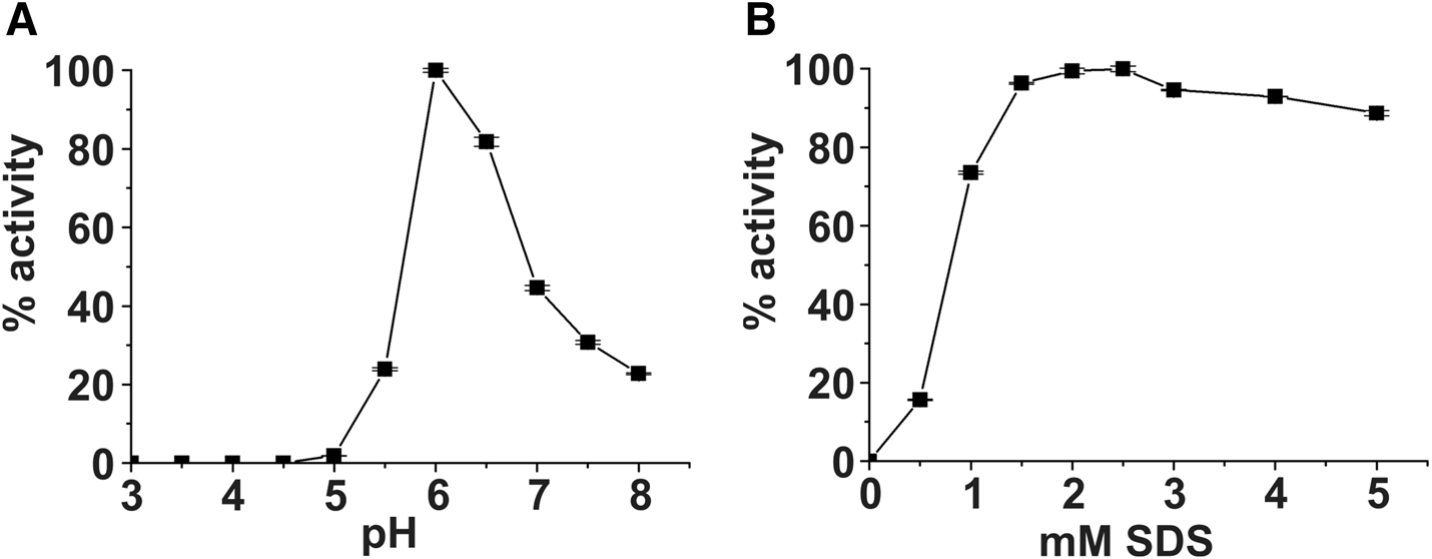
The pH (**A**) and SDS (**B**) profile of *jr*PPO2. The error bars indicate ± one standard deviation. Activities towards the standard substrate dopamine are plotted in relation to the maximum activity set to 100%. Measurements were performed in triplicates. Detailed information about the experimental setup is provided in the materials and methods section.

*jr*PPO2 was characterized in terms of its pH optimum and activation by SDS using 1 mM dopamine (Fig. S8) as a substrate. Different pH values for maximum activity have been reported for plant PPOs ranging from pH 4.5 (sodium citrate buffer)^48^ to pH 8.0 (sodium phosphate buffer)^49^. Thus, the pH dependence was assessed in increments of 0.5 pH units ranging from pH 3.0 to pH 8.0 (pH 3.0–pH 5.5: sodium citrate buffer, pH 6.0–pH 8.0: sodium phosphate buffer). The maximum activity was observed at pH 6.0 (Fig. 2), which follows the pH optimum of *jr*PPO1 (pH 6.0)^10^.

A general characteristic of plant PPOs is their latency^50^ as activity can be measured only in the presence of an additional activator. SDS has been proven suitable in activating plant PPOs^10,21,51^ and was previously shown to overcome their latency. Thus, the activation of PPOs is achieved with SDS molarities ranging from 0.35 mM^51^ to 4.0 mM^21^. We tested the concentration-dependent activation of *jr*PPO1 with SDS molarities ranging from 0.5 to 5.0 mM. The highest activity was observed at 2.5 mM SDS (Fig. 2), compared to 2.0 mM for *jr*PPO1^10^. The respective pH optima and SDS optima of *jr*PPO1 and *jr*PPO2 were used for substrate scope assays and the kinetic measurements.

*jr*PPO2 was characterized kinetically using the monophenolic substrates tyramine and l-tyrosine and the diphenolic substrates dopamine and l-DOPA (Fig. S8). *jr*PPO2 showed activity towards both monophenolic and both diphenolic substrates and, therefore, was classified as a TYR. *k*_cat_ (s^−1^) and *K*_m_ (mM) values were determined for tyramine, l-tyrosine, dopamine and l-DOPA (Table 2).

**Table 2:** Enzymatic parameters of standard substrates and natural substrates for *jr*PPO1, *jr*PPO2, *jr*PPO1-Asn240Gly and *jr*PPO2-Gly240Asn.

Substrate	Enzyme	*k*_cat_ (s^−1^)	*K*_m_ (mM)	*k*_cat_/*K*_m_ (s^−1^/mM)
Tyramine	*jr*PPO1	24.7 ± 1.50^†^	0.45 ± 0.08^†^	54.9 ± 11.0^†^
	*jr*PPO2	9.14 ± 0.83	0.49 ± 0.11	18.7 ± 4.65
	*jr*PPO1-Asn240Gly	7.60 ± 0.51	0.55 ± 0.10	13.8 ± 2.68
	*jr*PPO2-Gly240Asn	10.9 ± 0.71	0.21 ± 0.06	51.9 ± 15.3
l-Tyrosine	*jr*PPO1	6.00 ± 1.10^†^	1.42 ± 0,37^†^	4.22 ± 1.40^†^
	*jr*PPO2	*	*	0.69 ± 0.01
Dopamine	*jr*PPO1	92.5 ± 7.80^†^	0.75 ± 0.13^†^	123 ± 24.0^†^
	*jr*PPO2	186 ± 19.1	0.79 ± 0.17	235 ± 56.1
	*jr*PPO1-Asn240Gly	300 ± 16.5	0.49 ± 0.07	612 ± 93.7
	*jr*PPO2-Gly240Asn	66.3 ± 5.20	0.50 ± 0.10	133 ± 28.5
l-DOPA	*jr*PPO1	111 ± 8.80^†^	6.20 ± 1.00^†^	17.9 ± 3.40^†^
	*jr*PPO2	132 ± 7.05	5.66 ± 0.70	23.3 ± 3.14
Pyrogallol	*jr*PPO1	23.0 ± 0.79	1.20 ± 0.13	19.2 ± 2.18
	*jr*PPO2	138 ± 5.13	1.00 ± 0.12	138 ± 17.3
Protocatechuic acid	*jr*PPO1	38.4 ± 2.29	9.73 ± 1.31	3.95 ± 0.58
	*jr*PPO2	9.84 ± 0.52	0.09 ± 0.02	109 ± 25.0
Gallic acid	*jr*PPO1	*	*	*
	*jr*PPO2	2.07 ± 0.18	0.18 ± 0.04	11.5 ± 2.74
Ethyl gallate	*jr*PPO1	0.72 ± 0.12	13.4 ± 3.34	0.05 ± 0.02
	*jr*PPO2	59.5 ± 3.89	6.60 ± 0.79	9.02 ± 1.22
4-Hydroxyphenylacetic acid	*jr*PPO1	38.1 ± 2.45	44.2 ± 5.85	0.86 ± 0.13
	*jr*PPO2	32.3 ± 2.18	4.43 ± 0.55	7.29 ± 1.03
Coumaric acid	*jr*PPO1	0.48 ± 0.02	0.44 ± 0.06	1.09 ± 0.16
	*jr*PPO2	0.37 ± 0.02	0.94 ± 0.12	0.39 ± 0.05
Caffeic acid	*jr*PPO1	3.98 ± 0.16	1.51 ± 0.16	2.64 ± 0.30
	*jr*PPO2	8.37 ± 0.20	0.21 ± 0.02	39.9 ± 3.91
Quercetin	*jr*PPO1	*	*	0.33 ± 0.04
	*jr*PPO2	*	*	0.88 ± 0.06
Taxifolin	*jr*PPO1	1.04 ± 0.04	0.79 ± 0.05	1.32 ± 0.10
	*jr*PPO2	19.6 ± 2.04	0.92 ± 0.15	21.3 ± 4.12

Values are reported ± one standard deviation.

*Represents parameters that could not be measured due to low activity and limited substrate solubility.

^†^Indicates values previously reported^10^ and added to this Table. *K*_m_ and *k*_cat_ values were determined as described in the materials and methods section.

*k*_cat_ values were higher for the diphenols (*k*_cat_ dopamine = 186 s^−1^; *k*_cat_ l-DOPA = 132 s^−1^) compared to the monophenols (*k*_cat_ tyramine = 9.14 s^−1^; Table 2). Moreover, the catalytic efficiency (*k*_cat_/*K*_m_) of *jr*PPO2 was considerably higher for the less polar substrate tyramine (*k*_cat_/*K*_m_ = 18.7 s^−1^ mM^−1^) compared to the carboxylic substrate L-tyrosine (*k*_cat_/*K*_m_ = 0.69 s^−1^ mM^−1^), which held also true for the diphenolic substrates (Table 2).

### A substrate scope assay shows varying substrate scopes for *jr*PPO1 and *jr*PPO2

The activity of recombinantly expressed *jr*PPO1 and *jr*PPO2 towards 16 aglyconic, phenolic compounds naturally present in walnut^29–33^ (Table S2 and S3) was tested. Eleven small phenolic compounds (pyrogallol, 4-hydroxybenzoic acid, protocatechuic acid, gallic acid, salicylic acid, vanillic acid, ethyl gallate, 4-hydroxyphenylacetic acid, coumaric acid, caffeic acid and ferulic acid; Fig. S4), four flavonoids (kaempferol, quercetin, taxifolin, myricetin; Fig. S5) and the naphthoquinone juglone (Fig. S5) were tested.

**Figure 3:**
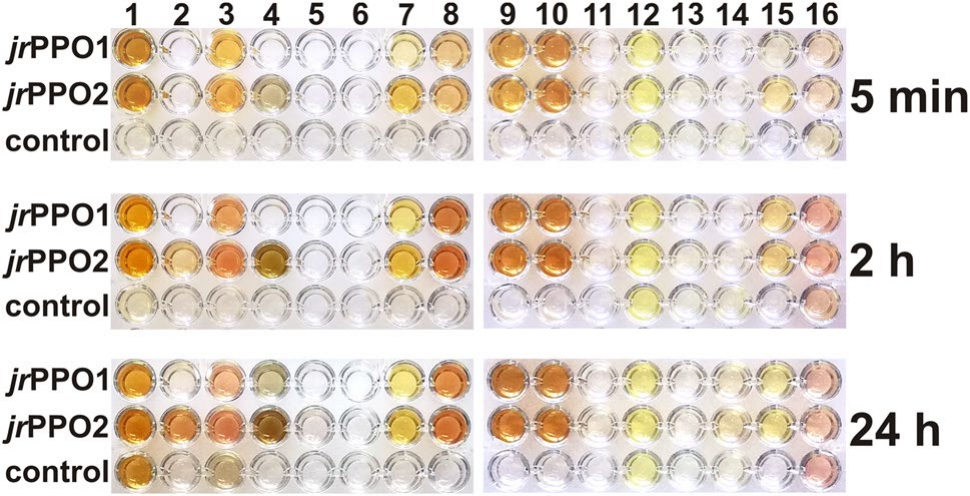
Substrate scope assay of *jr*PPO1 and *jr*PPO2 including 16 natural substrates: 1 = pyrogallol, 2 = 4-hydroxybenzoic acid, 3 = protocatechuic acid, 4 = gallic acid, 5 = salicylic acid, 6 = vanillic acid, 7 = ethyl gallate, 8 = 4-hydroxyphenylacetic acid, 9 = coumaric acid, 10 = caffeic acid, 11 = ferulic acid, 12 = juglone, 13 = kaempferol, 14 = quercetin, 15 = taxifolin, 16 = myricetin (Figs. S4 and S5). The control lane contained no enzyme. Photos were taken after 5 minutes, 2 hours and 24 hours and edited using GIMP 2.10.18 (https://www.gimp.org). Detailed information about the experimental setup is provided in the materials and methods section.

Substrate-enzyme combinations leading to a visually detectable change in color within 24 hours were flagged as active, whereas substrate-enzyme combinations remaining colorless after this time were flagged as inactive (Fig. 3).

Nine substrates were accepted by both enzymes (*jr*PPO1 and *jr*PPO2): pyrogallol, protocatechuic acid, gallic acid, ethyl gallate, 4-hydroxyphenylacetic acid, coumaric acid, caffeic acid, quercetin and taxifolin (Figs. S4 and S5) showed a clearly visible change in color within 24 hours (Fig. 3). Substrates carrying a 3-methoxy group (vanillic acid and ferulic acid) were rejected by both enzymes (*jr*PPO1 and *jr*PPO2), in contrast to their non-methoxylated homologs (vanillic acid / 4-hydroxybenzoic acid and ferulic acid/coumaric acid) (Fig. 3). Consequently, the 3-methoxy group is incompatible with the enzymatic activity of *jr*PPO1 and *jr*PPO2. Moreover, salicylic acid, which carries a 2-hydroxy group, kaempferol, myricetin and the naphthoquinone juglone were rejected by both *jr*PPO1 and *jr*PPO2 (Fig. 3). Several substrates showed varying reaction rates for *jr*PPO1 compared to *jr*PPO2, however, the differences were most prominent for the two benzoic acid derivatives protocatechuic acid and gallic acid. Protocatechuic acid (Fig. S4) was accepted by *jr*PPO2 (after ~ 2 hours) but rejected by *jr*PPO1 (after 24 hours). Similarly, gallic acid (Fig. S4) was oxidized by *jr*PPO2 within minutes, whereas activity towards *jr*PPO1 was detected only after 24 hours (Fig. 3).

### Kinetic measurements of *jr*PPO1 and *jr*PPO2 identify different substrate preferences

To further investigate the kinetic behavior of *jr*PPO1 and *jr*PPO2, *k*_cat_ and *K*_m_ values were determined for substrates that showed activity towards *jr*PPO1 and/or *jr*PPO2. Molar extinction coefficients were reported previously^52^ or were determined herein (see supplementary information; Table S2). The flavonoid substrates (quercetin and taxifolin; Fig. S5) were assayed in a solution containing 10% DMSO due to their limited water solubility. The effects of 10% DMSO on the activities of *jr*PPO1 and *jr*PPO2 were assessed using dopamine. In the presence of 10% DMSO, *jr*PPO1 retained 72% activity and *jr*PPO2 retained 75% activity, compared to enzymatic tests without additional DMSO. Thus, both enzymes are similarly affected by the addition of 10% DMSO.

*jr*PPO1 and *jr*PPO2 were more active towards diphenols than towards the corresponding monophenols (Table 2), as in general reported for PPOs^10,17,21,44^. However, *jr*PPO2 showed a stronger preference for diphenols over monophenols than *jr*PPO1, as diphenolic and triphenolic substrates showed higher activity values (*k*_cat_ value) and higher efficiency values (*k*_cat_/*K*_m_ ratio) towards *jr*PPO2 than towards *jr*PPO1. The only exception was protocatechuic acid (Fig. S4), which was more active (higher *k*_cat_ value) with *jr*PPO1. However, since the *K*_m_ value for protocatechuic acid (Fig. S4) increased for *jr*PPO1 (compared to *jr*PPO2), it showed a substantially higher catalytic efficiency towards *jr*PPO2 (*k*_cat_/*K*_m_ = 109 s^−1^ mM^−1^), compared to *jr*PPO1 *(k*_cat_/*K*_m_ = 3.95 s^−1^ mM^−1^) (Table 2). In contrast, all monophenolic substrates (4-hydroxyphenylacetic acid, coumaric acid, l-tyrosine, and tyramine) showed a higher turnover rate towards *jr*PPO1, compared to *jr*PPO2. Monophenolase/diphenolase activity ratios (*k*_cat_ monophenol/*k*_cat_ diphenol) of corresponding mono- and diphenols were higher for *jr*PPO1 than for *jr*PPO2. The activity ratio of tyramine/dopamine for *jr*PPO1 was 0.27, compared to 0.05 for *jr*PPO2 (Table 2). The same trend held true for the monophenolase/diphenolase efficiency ratios ((*k*_cat_/*K*_m_) monophenol/(*k*_cat_/*K*_m_) diphenol).

Thus, our data show that *jr*PPO1 favors monophenolic substrates, whereas *jr*PPO2 targets diphenolic substrates. This trend also correlates with the flavonoid substrates and was particularly pronounced for the diphenolic substrate taxifolin (Fig. S5), which was 19-times more active towards *jr*PPO2 (*k*_cat_ = 19.6 s^−1^), compared to *jr*PPO1 (*k*_cat_ = 1.04 s^−1^; Table 2). An asparagine in the position of the 1st activity controller residue (Asn240) has previously been proven to increase monophenolase activity^10,25^, which explains the higher activity (k_cat_ values) of *jr*PPO1 towards monophenols, compared to *jr*PPO2. To clarify the molecular cause for the increased diphenolase activity of *jr*PPO2, compared to *jr*PPO1, docking studies were employed.

### Docking studies illustrate the molecular cause of the different reactivities of *jr*PPO1 and *jr*PPO2

For the docking studies, a homology model of *jr*PPO2 was built using the SWISS-MODEL server^53,54^ (Fig. S3) and the crystal structure of *jr*PPO1 (PDB entry 5CE9) as a template. Molecular docking was performed for *jr*PPO1 as well as *jr*PPO2. Binding poses were calculated for all kinetically investigated substrates, which included the standard substrates tyramine, l-tyrosine, dopamine and l-DOPA and the natural substrates pyrogallol, protocatechuic acid, gallic acid, ethyl gallate, 4-hydroxyphenylacetic acid, coumaric acid, caffeic acid, quercetin and taxifolin. The results offered highly valuable information detailing the molecular basis for the different reactivities of *jr*PPO1 and *jr*PPO2.

The homology model of *jr*PPO2 exhibited a high level of structural homology, compared to the crystal structure of *jr*PPO1 (RMSD = 0.487 Å). However, the amino acid in the position of the 1st activity controller residue represents a notable difference between the architectures of the active centers of *jr*PPO1 and *jr*PPO2 (Figs. S2 and S3). *jr*PPO1 features a Gly in this position, whereas *jr*PPO2 features a spatially more demanding Asn, which is protruding directly into the active center (Fig. S9).

**Figure 4:**
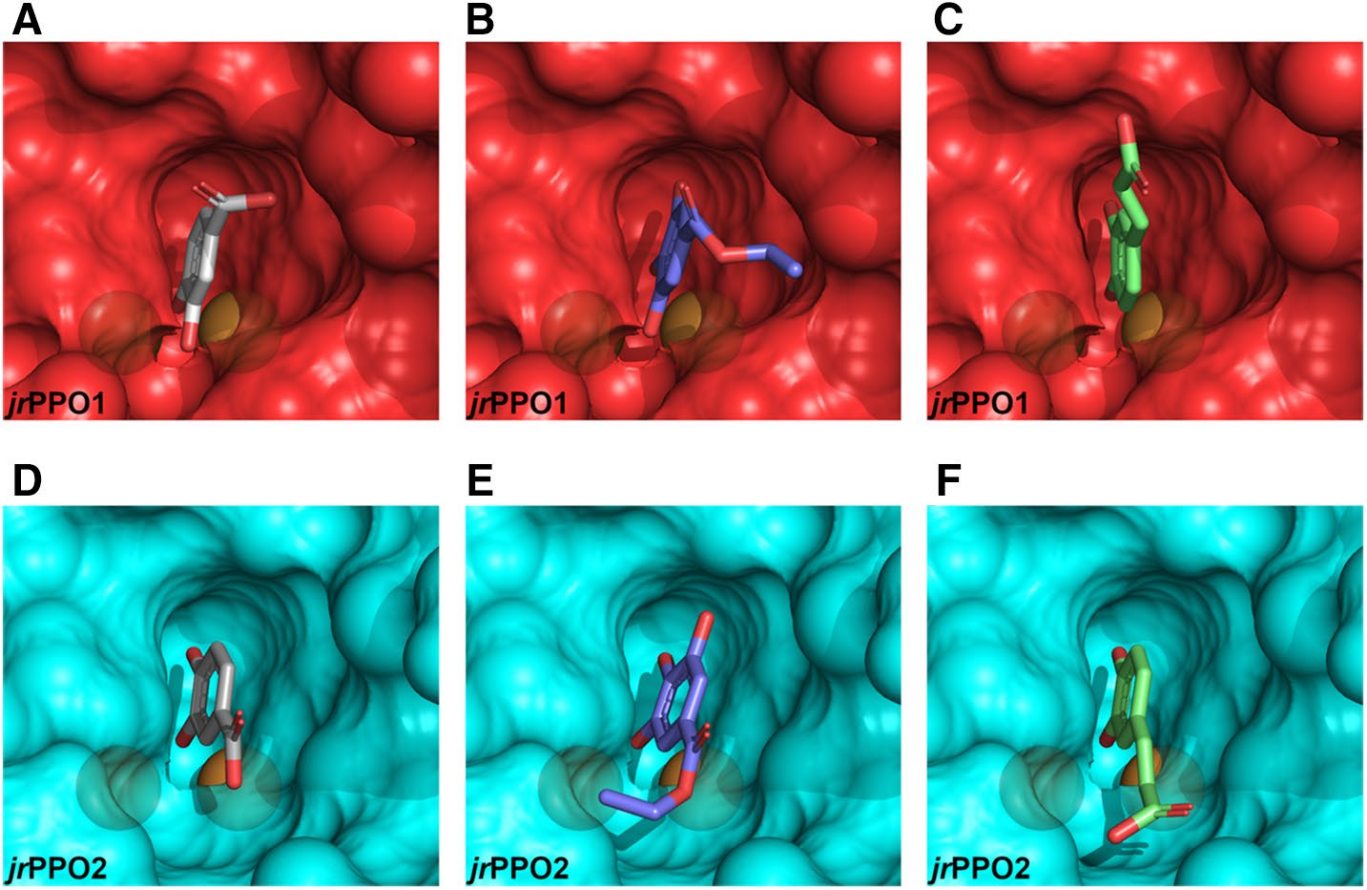
Docking poses of protocatechuic acid, ethyl gallate, and caffeic acid. The transparency was set to 0.3, the copper ions are displayed as brown spheres. (**A**–**C**) (red) represent the active center of *jr*PPO1. (**D**–**F**) (blue) represent the active center of *jr*PPO2, with substrates docked as follows: A = protocatechuic acid, B = ethyl gallate, C = caffeic acid, D = protocatechuic acid, E = ethyl gallate, F = caffeic acid. The active centers of *jr*PPO1 and *jr*PPO2 are shown as seen by incoming substrates. The images were created using PyMOL 2.3^58^ and edited using GIMP 2.10.18 (https://www.gimp.org).

The calculated binding poses clearly show that in *jr*PPO2 all diphenolic substrates are preferentially oriented in a lying down position (orienting the 3′-hydroxy group toward the di-copper center; Figs. 4, S10, S11), whereas, in *jr*PPO1, diphenolic substrates have to approach the di-nuclear center in an upright orientation (orienting the 4′-hydroxy group toward the di-copper center; Fig. 4). Orienting diphenolic substrates in a lying down position in *jr*PPO1 is prevented by Asn240, which overlaps with the tails of diphenolic substrates in *jr*PPO2. Alternatively, diphenolic substrates can be oriented in *jr*PPO2 in an upright position as well (data not shown). Thus, orienting substrates into the active center of *jr*PPO2 with the phenolic ring facing the di-copper center appears to be entropically more favorable, compared to *jr*PPO1, due to the spatially less demanding 1st activity controller residue (Gly240). This explains the significantly higher turnover rates and efficiency values of diphenolic substrates for *jr*PPO2, compared to *jr*PPO1 (Table 2).

**Figure 5:**
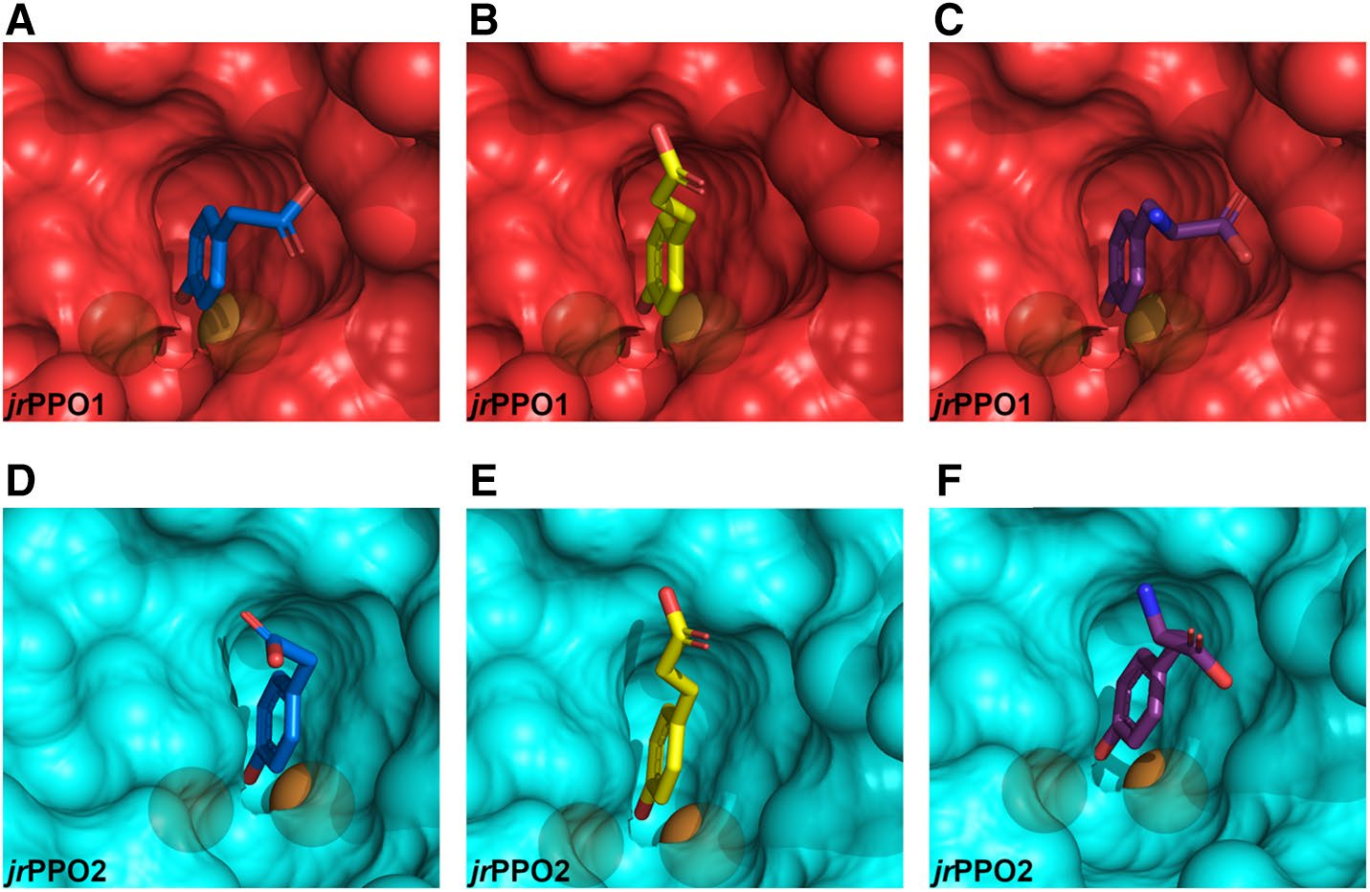
Docking poses of 4-hydroxyphenylacetic acid, coumaric acid, and tyrosine. The transparency was set to 0.3, the copper ions are displayed as brown spheres. (**A**–**C**) (red) represent the active center of *jr*PPO1. (**D**–**F**) (blue) represent the active center of *jr*PPO2, with substrates docked as follows: A = 4-hydroxyphenylacetic acid, B = coumaric acid, C = tyrosine, D = 4-hydroxyphenylacetic acid, E = coumaric acid, F = tyrosine. The active centers of *jr*PPO1 and *jr*PPO2 are viewed as seen by incoming substrates. The images were created using PyMOL 2.3^58^ and edited using GIMP 2.10.18 (https://www.gimp.org).

In contrast, monophenolic substrates featuring a 4′-hydroxy group are oriented exclusively in an upright position in *jr*PPO1 and *jr*PPO2, as demonstrated by the molecular docking poses (Figs. 5, S10, S11). Thus, the entropic advantage of the more spacious active center of *jr*PPO2 does not come into effect for monophenolic substrates. Moreover, the asparagine present in the position of the 1st activity controller in *jr*PPO1 has been shown to facilitate monophenolase activity by aiding in the imperative abstraction of the phenolic proton from incoming monophenolic substrates^20^. The resulting phenolate substrate, carrying a negative charge, exhibits an increased affinity towards the positively charged di-copper center, compared to the corresponding not-dissociated phenol^20^. This was first demonstrated for *Vv*PPOcs-3^25^, which features a glycine in the position of the 1st activity controller residue (Gly241). Semiquantitative in-gel activity tests demonstrated that the mutant *Vv*PPOcs-3-Gly241Asn (1st activity controller: Asn241) showed increased activity rates towards the monophenolic substrates tyramine and p-tyrosol (compared to the native enzyme; 1st activity controller: Gly241)^25^, which is in accordance with our results. Moreover, it has been demonstrated, based on the crystal structure of the bacterial tyrosinase from *Bacillus megaterium* (*Bm*TYR)^55^, which also features an Asn in the position of the 1st activity controller (His_B1_ + 1 = Asn205), that Asn205 forms a polar bond with the first CuB coordinating histidine (His_B1_ = His204) and, thereby, stabilizes His204^55^. In *Bm*TYR, the N_δ1_ atom of the imidazole ring of His204 (His_B1_) is located at a distance of 2.7 Å from the amide group of Asn204 (1st activity controller residue). Similarly, the N_δ1_ atom of the imidazole ring of His239 (His_B1_) in *jr*PPO1 is located at a distance of 2.9 Å from the amide group of Asn240 (1st activity controller residue)^9^. Thus, in *jr*PPO1 Asn240 probably shows a stabilizing effect on His239. The combination of these effects explains the higher activity rates of monophenolic substrates with *jr*PPO1, compared to *jr*PPO2 (Table 2).

### Mutagenesis studies confirm the pivotal influence of the 1st activity controller on enzymatic activity

**Figure 6:**
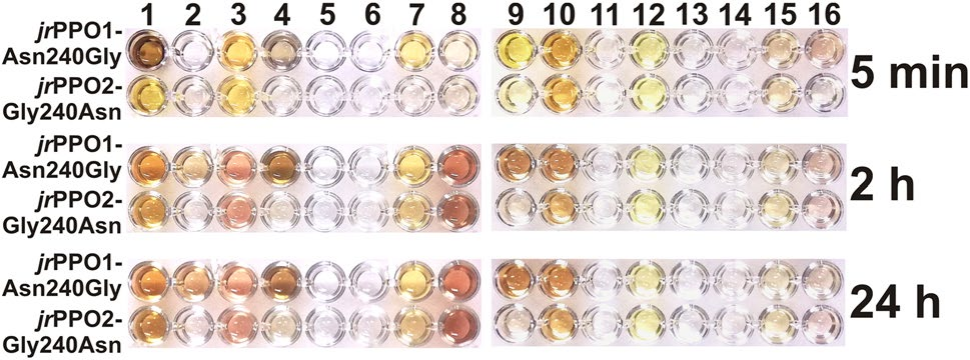
Substrate scope assay of *jr*PPO1-Asn240Gly and *jr*PPO2-Gly240Asn including 16 natural substrates: 1 = pyrogallol, 2 = 4-hydroxybenzoic acid, 3 = protocatechuic acid, 4 = gallic acid, 5 = salicylic acid, 6 = vanillic acid, 7 = ethyl gallate, 8 = 4-hydroxyphenylacetic acid, 9 = coumaric acid, 10 = caffeic acid, 11 = ferulic acid, 12 = juglone, 13 = kaempferol, 14 = quercetin, 15 = taxifolin, 16 = myricetin (Figs. S4 and S5). Photos were taken after 5 minutes, 2 hours and 24 hours and edited using GIMP 2.10.18 (https://www.gimp.org). Detailed information about the experimental setup is provided in the materials and methods section.

The mutants *jr*PPO1-Asn240Gly and *jr*PPO2-Gly240Asn were generated by site-directed mutagenesis (Table S1) to further prove the influence of the amino acid residue present in the position of the 1st activity controller. Now, in the position of the 1st activity controller, *jr*PPO1-Asn240Gly resembles *jr*PPO2, whereas *jr*PPO2-Gly240Asn resembles *jr*PPO1. A substrate scope assay revealed the preferences of each mutant towards natural substrates and proved that *jr*PPO1-Asn240Gly resembles *jr*PPO2 in terms of substrate preferences as it accepts 4-hydroxybenzoic acid and showed activity with gallic acid (Fig. S4) and ethyl gallate (Fig. S4) within minutes (Figs. 3 and 6). In contrast, *jr*PPO2-Gly240Asn rejected 4-dihydroxybenzoic acid, which was rejected by *jr*PPO1 but accepted by *jr*PPO2. Gallic acid and ethyl gallate were both oxidized by *jr*PPO2-Gly240Asn after several hours, which corresponds to the substrate scope assay of *jr*PPO1 (Figs. 3 and 6).

Furthermore, kinetic parameters were determined for the monophenolic substrate tyramine and the diphenolic substrate dopamine for both mutants (Table 2). In accordance with our previous results, *jr*PPO1-Asn240Gly showed a considerably increased activity towards the diphenol dopamine, compared to *jr*PPO1 (*k*_cat_ *jr*PPO1-Asn240Gly = 300 s^−1^, *k*_cat_ *jr*PPO1 = 92.5 s^−1^), whereas the activity towards the monophenol tyramine was reduced (*k*_cat_ *jr*PPO1-Asn240Gly = 7.60 s^−1^, *k*_cat_ *jr*PPO1 = 24.7 s^−1^) (Table 2). In contrast, *jr*PPO2-Gly240Asn showed a reduced activity towards dopamine (*k*_cat_ *jr*PPO2-Gly240Asn = 66.3 s^−1^, *k*_cat_ *jr*PPO2 = 186 s^−1^) and an increased activity towards tyramine (*k*_cat_ *jr*PPO2-Gly240Asn = 10.9 s^−1^, *k*_cat_ *jr*PPO2 = 9.14 s^−1^). Accordingly, *jr*PPO1-Asn240Gly (tyramine/dopamine activity ratio = 0.03) had a stronger preference of diphenols over monophenols than *jr*PPO2-Gly240Asn (tyramine/dopamine activity ratio = 0.16; Table 2).

**Figure 7:**
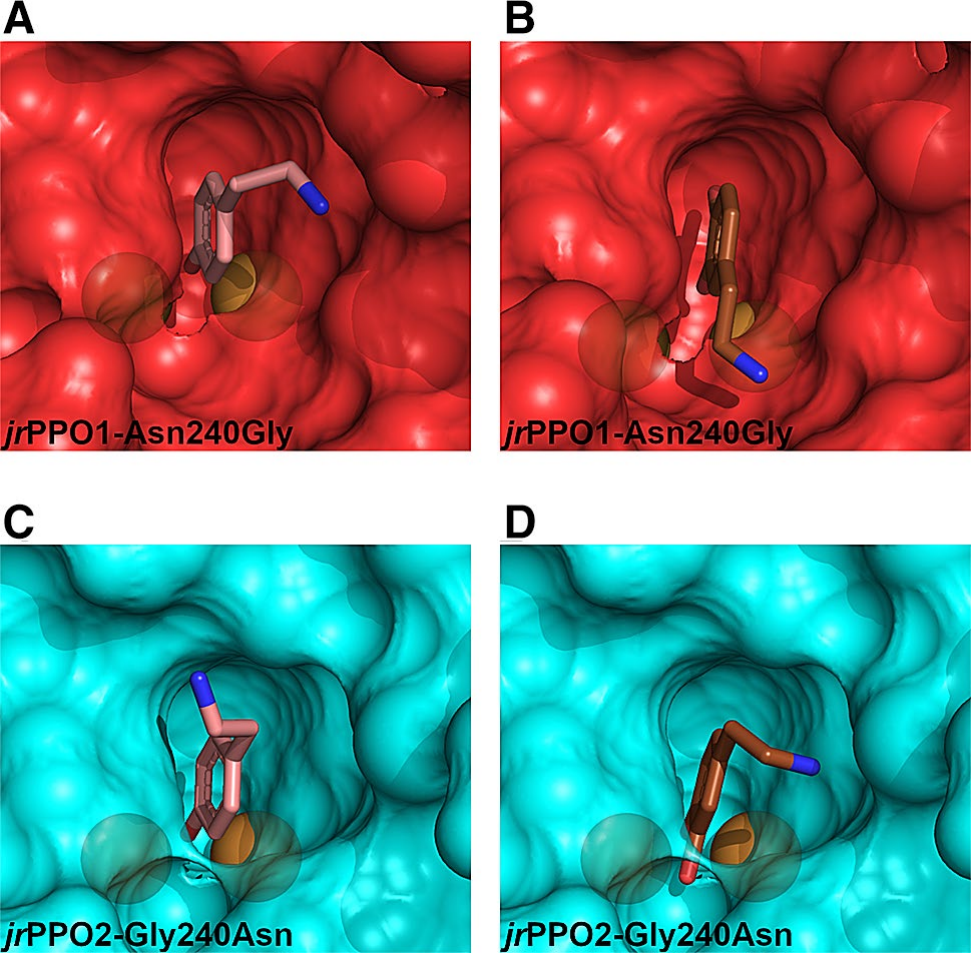
Docking poses calculated for diphenolic substrates for *jr*PPO1-Asn240Gly and *jr*PPO2-Gly240Asn. The transparency was set to 0.3. The copper ions are displayed as brown spheres. (**A**, **B**) (red) represent the active center of *jr*PPO1-Asn240Gly. (**C**, **D**) (blue) represent the active center of *jr*PPO2-Gly240Asn, with substrates docked as follows: A = tyramine, B = dopamine, C = tyramine, D = dopamine. The active centers of *jr*PPO1-Asn240Gly and *jr*PPO2-Gly240Asn are viewed as seen by incoming substrates. The images were created using PyMOL 2.3^58^ and edited using GIMP 2.10.18 (https://www.gimp.org).

Docking studies were performed for *jr*PPO1-Asn240Gly und *jr*PPO2-Gly240Asn using tyramine and L-tyrosine (Fig. 7) and all substrates investigated during the previous docking experiments (Figs. 4, 5, S10–S13). Our data show that diphenolic substrates are oriented in a laying down position in *jr*PPO1-Asn240Gly (as observed for *jr*PPO2; Figs. 4, 7, S12 and S13). In contrast, in *jr*PPO2-Gly240Asn diphenolic substrates must approach the di-nuclear copper center in an upright position, since Asn240 now blocks substrates from orienting in the laying down orientation (Figs. 7, S12 and S13). Monophenolic substrates enter the active center of both mutants (*jr*PPO1-Asn240Gly and *jr*PPO2-Gly240Asn) in an upright orientation.

This data show that the amino acid residue in the position of the 1st activity controller is responsible for the different substrate preferences of *jr*PPO1 (targeting monophenols) and *jr*PPO2 (targeting diphenols). The substrate scope is dependent on the amino acid present in the position of the 1st activity controller. Moreover, an asparagine in the position of the 1st activity controller increases monophenolase activity, whereas diphenolase activity is reduced (compared to the presence of glycine in the same position). Substituting asparagine with a spatially less demanding amino acid (such as glycine) increased the *k*_cat_ value of dopamine considerably. Our results lead to the conclusion that *jr*PPO1 and *jr*PPO2 in vivo target different substrates and, thus, most probably fulfill different physiological tasks. The common appearance of plant PPOs as a family of isoenzymes suggests that they are involved in several cellular pathways, covering a diverse spectrum of functionalities. We hope that our work will inspire the deciphering of the different tasks assigned to PPOs, thereby illuminating their elusive reactivities.

## Conclusions

In this study, *jr*PPO2 was, for the first time, heterologously expressed, purified and characterized. Activity tests using standard substrates (tyramine, l-tyrosine, dopamine, l-DOPA) clarified that *jr*PPO2 is a TYR, as it accepted l-tyrosine and tyramine. Moreover, substrate scope assays using 16 natural substrates showed a more expansive substrate scope for *jr*PPO2 as it accepted 4-hydroxybenzoic acid and gallic acid, compared to *jr*PPO1, which was inactive with 4-hydroxybenzoic acid and showed only marginal activity towards gallic acid. Kinetic parameters were determined for *jr*PPO2 and its isoenzymes *jr*PPO1, which pointed towards differences in substrate preference. *jr*PPO2 showed a higher catalytic efficiency for diphenols whereas *jr*PPO1 was more active on monophenols. Docking studies revealed that the amino acid in the position of the 1st activity controller can increase the activity towards monophenolic substrates, as it has previously been proposed, by stabilizing a conserved water molecule or reduce enzymatic activity towards diphenolic substrates by sterically impeding substrate orientation. The two mutants *jr*PPO1-Asn240Gly and *jr*PPO2-Gly240Asn proved the key role of the 1st activity controller as *jr*PPO1-Asn240Gly showed an enzymatic profile similar to *jr*PPO2, whereas *jr*PPO2-Gly240Asn resembled *jr*PPO1.

Our results demonstrate that, in vivo, different PPOs within the same plant target different substrates, which is achieved by the variability of one crucial amino acid residue (1st activity controller). This novel understanding of the functionality of PPO isoenzymes in plants will hopefully allow controlling their reactivity and, thereby, enhance the nutritional and economic value of plant products.

## Materials and methods

### Isolation of genomic DNA and cloning of the *jr*PPO2 gene

Walnut leaves were collected from naturally grown trees around Vienna and stored at -80 °C. Two g of frozen leaves were ground in liquid nitrogen. The frozen paste was mixed with 2 ml extraction buffer (100 mM HEPES, 0.5 M NaCl, 20 mM sodium ascorbate, 2% PVP and 1% cetyltrimethylammonium bromide (CTAB), pH 8.0)^42^. The mixture was incubated in a 70 °C water bath for one hour followed by centrifugation at 20.000 rpm for 10 min. The supernatant was extracted with 1 volume of phenol:chloroform:isoamyl alcohol (25:24:1, pH 7.8) and subsequently the aqueous layer was washed two times with 1 volume 100% chloroform. The aqueous layer was precipitated by adding 1 volume of EtOH (96%) and incubation at 0 °C for 2 hours. The pellet resulting from centrifugation at 20.000 rpm for 10 min at 4 °C was washed two times with EtOH (70%) at 0 °C, dried and resuspended in 100 µl TE buffer (10 mM Tris—HCl, 1 mM EDTA, pH 8.0). The quality and quantity of the DNA extract were checked by 0.6% agarose gel electrophoresis (Fig. S14).

The first pair of primers binding outside of the open reading frame of the active domain and the C-terminal domain was designed (using the NEB Tm calculator v1.12.0) from the sequence of *jr*PPO2 published previously^19^ (Table S1). *Q5 High-Fidelity DNA polymerase* (NEB, Ipswich, USA) was used for the amplification and a ~ 1,700 base pair amplicon was produced (for detailed PCR setup see supplementary information). The PCR product was cloned into the pENTRY-IBA51 vector and sequenced to reveal the full-length sequence of *jr*PPO2. Thereafter, the gene encoding *jr*PPO2 (active and C-terminal domain) was amplified with the second pair of primers (designed based on the sequencing results; Table S1). Using *Q5 High-Fidelity DNA polymerase* a ~ 1,500 base pair amplicon was obtained, cloned into the pENTRY-IBA51 vector and again sequenced. The sequence-verified construct was sub-cloned into the open reading frame of a pGEX-6P-1 based expression vector using the *Esp3I* restriction enzyme (Thermo Fisher, Waltham, USA) and transformed into *E. coli* BL21 (DE3) cells.

### Construction of the mutants *jr*PPO1-Asn240Gly and *jr*PPO2-Gly240Asn

The genes encoding *jr*PPO1 and *jr*PPO2, cloned into the pENTRY-IBA51 donor vector, respectively, served as templates for the mutagenesis experiments. *Q5 High-Fidelity DNA polymerase* was used to introduce the mutations into the sequence by back to back annealing primers (Table S1) with the forward primer carrying the desired mutation. *T4 Polynucleotide Kinase* (NEB) and *T4 DNA Ligase* (NEB) were used to create cyclic plasmids (pENTRY-IBA51). The open reading frames were then sub-cloned into the pGEX-6P-1 expression vector using the *Esp3I* restriction enzyme and expressed as described previously^10^ (see supplementary information).

### Molecular mass determination via mass spectrometry

Mass spectra of *jr*PPO1, *jr*PPO2, *jr*PPO1-Asn240Gly and *jr*PPO2-Gly240Asn were measured on an LTQ Orbitrap Velos mass spectrometer (Thermo Fisher Scientific, Bremen, Germany) equipped with a nanospray ion source using an ion transfer capillary temperature of 300 °C and an electrospray voltage of 2.1 kV. 5 µl of the sample was loaded on a trap column of an UltiMate 3000 nano HPLC-system (Dionex) using 0.1% trifluoroacetic acid. The separation was carried out at a flow rate of 300 nl/min on a C4 analytical column 50 cm × 75 µm Accucore C4, 150 Å, 2.6 µm (Thermo Fisher Scientific) using mobile phase A (2% acetonitrile, 0.1% formic acid and 98% H_2_O) and mobile phase B (0.1% formic acid, 20% H_2_O and 80% acetonitrile). Full MS scans were acquired in positive ion mode ranging from 400 to 2000 m/z at a resolution of 7,500 (FWHM at 400 m/z).

### Characterization of *jr*PPO2, substrate scope assays and kinetic investigation of *jr*PPO1, *jr*PPO2, *jr*PPO1-Asn240Gly and *jr*PPO2-Gly240Asn

Kinetic measurements were performed in triplicates. Photometric measurements were all carried out on TECAN infinity M200 (Tecan, Salzburg, Austria) in 96 well plates at 25 °C using the latent enzyme and SDS as an activator. pH and SDS optima were determined for *jr*PPO2 using the diphenolic substrate dopamine. The highest activities were measured at pH 6.0 (50 mM sodium phosphate buffer) and 2.5 mM SDS. Kinetic measurements of *jr*PPO2 and *jr*PPO2-Gly240Asn were performed under these conditions by measuring the increase of the colored reaction products photometrically (Fig. S19). For *jr*PPO1 the optimal conditions were a pH value of 6.0 and 2.0 mM SDS, as published previously^10^. Identical conditions were used for the mutant *jr*PPO1-Asn240Gly.

To determine which substrates showed activity with *jr*PPO1 and/or *jr*PPO2, 100 µg of the purified, latent enzyme were mixed with 1 mM substrate in 50 mM sodium phosphate buffer and 2 mM (*jr*PPO1 and *jr*PPO1-Asn240Gly) or 2.5 mM (*jr*PPO2 and *jr*PPO2-Gly240Asn) SDS in 200 µl solution at 25 °C. Due to their limited solubility, the assays for the flavonoid substrates (kaempferol, quercetin, taxifolin, myricetin; Fig. S5) and juglone were performed using 0.1 mM substrate, 100 µg enzyme, 50 mM sodium phosphate buffer, 2 mM (*jr*PPO1 and *jr*PPO1-Asn240Gly) or 2.5 mM (*jr*PPO2 and *jr*PPO2-Gly240Asn) SDS and 10% DMSO in 200 µl at 25 °C. Substrates that exhibited no visually detectable change in color within 24 hours were flagged as inactive. A control assay was performed for each substrate containing 1 mM substrate in 50 mM sodium phosphate buffer and 2.5 mM SDS in 200 µl at 25 °C.

For calculating the kinetic parameters (*k*_cat_ and *K*_m_ value), the maximum reaction rate was measured at 7–8 different substrate concentrations in a total volume of 200 µl containing 50 mM sodium phosphate buffer at pH 6.0, 2 mM (*jr*PPO1 and *jr*PPO1-Asn240Gly) or 2.5 mM (*jr*PPO2 and *jr*PPO2-Gly240Asn) SDS and variable amounts of enzyme (Table S3). For quercetin and taxifolin, DMSO was added to a final concentration of 10% to increase the solubility of the substrates. The data were fitted to the Michaelis–Menten equation by non-linear curve fitting (OriginPro 8 software; Figs. S15–S18).

### Molecular docking with *jr*PPO1 and *jr*PPO2

Molecular docking was performed using AutoDock Vina^56^. The crystal structure of *jr*PPO1 (PDB entry 5CE9) was prepared for molecular docking by adding missing side chains using Coot^57^. A homology model of *jr*PPO2 was built using the SWISS-MODEL server^53,54^. The exhaustiveness was set to 100 and 20 poses were calculated for each target and substrate (Tables S4 and S5). Structures of the substrates were obtained from PubChem and formatted into pdbqt files using AutoDockTools (ADT, v.1.5.6)^56^. Docking studies were performed with protonated, semi-protonated and deprotonated hydroxy-phenyl groups (generated by editing the substrate pdbqt files). Binding poses were searched in a grid box enclosing the two copper ions of the active site, the 1st and 2nd activity controller and Phe260 (Figs. S1 and S2). For *jr*PPO1, *jr*PPO1-Asn240Gly, *jr*PPO2 and *jr*PPO2-Gly240Asn the 1st activity controller residue (*jr*PPO1 and *jr*PPO2-Gly240Asn: Asn240, *jr*PPO2 and *jr*PPO1-Asn240Gly: Gly240), the 2nd activity controller residue (*jr*PPO1 and *jr*PPO1-Asn240Gly: Leu244, *jr*PPO2 and *jr*PPO2-Gly240Asn: Ile244) and Phe260, were defined as flexible residues. Poses that significantly deviated from the binding pose of l-tyrosine were flagged as ’unreasonable’ poses. All visualizations were created using PyMOL 2.3^58^.

## Supplementary information

Supplementary information
