# Supplementary material for: Identification of the amino acid position controlling the different enzymatic activities in walnut tyrosinase isoenzymes (jrPPO1 and jrPPO2)
Source: Sci Rep. 2020 Jul 2;10:10813. doi: 10.1038/s41598-020-67415-6 (PMC7331820; doi:10.1038/s41598-020-67415-6)
Supplement: Supplementary file 1 — Supplementary information [file 41598_2020_67415_MOESM1_ESM.docx]

**Supplementary Information**

**Identification of the amino acid position controlling the different enzymatic activities in walnut tyrosinase isoenzymes (*jr*PPO1 and *jr*PPO2).**

Felix Panis^[1]^ and Annette Rompel*^[1]^

^1^Universität Wien, Fakultät für Chemie, Institut für Biophysikalische Chemie, Althanstraße 14, 1090 Wien, Austria; https://www.bpc.univie.ac.at

*Correspondence to: [*annette.rompel@univie.ac.at*](mailto:annette.rompel@univie.ac.at)

**Table of content**

1. Supplementary Materials and Methods 2
2. Supplementary Tables 4
3. Supplementary Figures 8
4. References 26

**1. Supplementary Materials and Methods**

**PCR experiments.** Primers were ordered from Merck (Darmstadt, Germany) and *Q5 High-Fidelity DNA polymerase, Deoxynucleotide (dNTP) Solution Mix* and *Q5 Reaction Buffer* were purchased from NEB (Ipswich, USA). 20 ng template DNA was mixed with 0.25 mM dNTPs, 0.2 units *Q5 High-Fidelity DNA polymerase* and 4 µl *5 x Q5 Reaction Buffer* in a total volume of 20 µl. After an initial denaturation step at 98 °C for 8 minutes DNA amplification was performed by 28 PCR cycles (15 seconds 98 °C, 20 seconds 65 °C, 90 seconds 72 °C) followed by a terminal elongation step at 72 °C for 6 minutes. The quantity and quality of the amplified DNA were assessed by 0.8 % agarose gel electrophoresis.

**Expression and purification of *jr*PPO1, *jr*PPO2, *jr*PPO1-Asn240Gly and *jr*PPO2-Gly240Asn.** 500 ml 2 x YT medium were inoculated with a freshly transformed colony and incubated at 37 °C until the OD_600_ reached a value of 1.2. Subsequently, 0.5 mM CuCl_2_ was added and the expression was induced using 0.5 mM isopropyl-β-D-thiogalactopyranoside (IPTG). Expression batches were incubated at 20 °C for 65 hours under shaking at 220 rpm. The same procedure was used for the expression of *jr*PPO1, *jr*PPO1-Asn240Gly and *jr*PPO2-Gly240Asn. The purification of all the investigated enzymes was performed according to a protocol previously reported for *jr*PPO1^1^. In short, the GST-tagged fusion proteins were separated from the cell lysate by a 5ml GST-rap FF column and subsequently, the GST-tag was cleaved by an in-house produced GST-HRV 3C protease^2^. After that, a second purification took place via the same column which trapped the cleaved GST-tag as well as the protease while the latent enzyme remained in the flow-through of the column.

The quantification of the protein was performed according to the Lambert-Beer law using an extinction coefficient calculated by the [ExPASy ProtParam tool](https://web.expasy.org/protparam/)^3^ and the absorption of the protein measured at 280 nm. The purity of the final protein was checked *via* SDS-PAGE under denaturing conditions.

As reported in a previous study^1^, *jr*PPO1 investigated herein contained the variation Val107Ile, compared to the sequence published by Escobar *et al.*^4^ and Martinez-Garcia *et al.*^5^, both of which originate from cultivated trees (cultivar “Chandler”) grown in California. In contrast, trees sampled in this study were naturally grown in the surroundings of Vienna, Austria. Thus, the different habitats account for the variation. No further mutations were detected in the *jr*PPO1 sequence, compared to previously published sequences.

The copper content was determined for *jr*PPO1, *jr*PPO2, *jr*PPO1-Asn240Gly and *jr*PPO2-Gly240Asn using a method published by Hanna *et al*., in which the formation of a copper-2,2′-biquinoline complex is quantified photometrically at 546 nm (ε = 6300 M^−1^ cm^−1^)^1,6^. Copper contents ranging from 0.9 - 1.2 copper atoms per active site were measured (*jr*PPO1: 0.9 ± 0.1, *jr*PPO2: 1.1 ± 0.1, *jr*PPO1-Asn240Gly: 1.0 ± 0.1, *jr*PPO2-Gly240Asn: 1.2 ± 0.1; the number of copper atoms per active site is presented ± one standard deviation), which is in accordance with previous copper content measurements of recombinantly expressed plant PPOs^1^. Thus, the copper content does not account for different enzymatic activities of *jr*PPO1, *jr*PPO2, *jr*PPO1-Asn240Gly and *jr*PPO2-Gly240Asn.

**Determination of molar extinction coefficients.** The molar extinction coefficients used herein for calculating *k*_cat_ values have been partially reported previously^7^ (dopamine, *L*-DOPA, pyrogallol, 4-hydroxyphenylacetic acid and caffeic acid). For the rest of the investigated substrates, extinction coefficients were determined within the scope of this study (protocatechuic acid, gallic acid, ethyl gallate, quercetin, taxifolin; Figures S4, S5 and S19). For monophenols, the extinction coefficients of the corresponding diphenols were used. The wavelength chosen represents the absorption maximum (quercetin, taxifolin) or an isosbestic point (since unstable quinones tend to react spontaneously over time)^7^.

Measurements were performed using a method described by Munoz *et al.*^7^. In short, sodium periodate (10 – 100-fold molar excess) was used to oxidize different substrate molarities to their corresponding quinones and the absorption values were plotted against the substrate concentrations to calculate a regression line using the OriginPro 8 software. For the flavonoid substrates (quercetin and taxifolin) 4 mM 3-methyl-2-benzothiazolinone hydrazone hydrochloride (MBTH, Besthorn’s hydrazone)^8^ was added to detect the formation of the quinones and 40 µM *jr*PPO2 was used to oxidize the substrates in 10 % DMSO. All measurements were performed in 50 mM sodium phosphate buffer pH 6.0 at 25 °C on a Shimadzu UV-1800 photometer.

The molar extinction coefficients of all substrates investigated kinetically, and their corresponding wavelengths are listed in Table S2.

**2. Supplementary Tables**

| *jr*PPO2* fwd | CTCCTGGAGCACTGTCATGCAGAGC |
| --- | --- |
| *jr*PPO2* rev | AGCCCGATTGGTCGATTAATAACGTACC |
| *jr*PPO2 fwd | GCTCCCGTATCAGCACCGGAC |
| *jr*PPO2 rev | ATCAAGAAGCTCAATCTTGATCCCATTAATGG |
| *jr*PPO1-Asn240Gly fwd | CCACggTAATATCCACCTATGGAC |
| *jr*PPO1-Asn240Gly rev | GGAGTGCTCTCGATTGTG |
| *jr*PPO2-Gly240Asn fwd | ACaaTCCTGTCCACATATGGAC |
| *jr*PPO2-Gly240Asn rev | GGGGAATGTTCTCTATAGAGCC |

**Table S1.** Primers used to obtain the full-length sequence of *jr*PPO2 (*jr*PPO2* fwd and *jr*PPO2* rev), primers used for obtaining the ORF of *jr*PPO2 (*jr*PPO2 fwd and *jr*PPO2 rev), primers used to create the mutant *jr*PPO1-Asn240Gly (*jr*PPO1-Asn240Gly fwd and *jr*PPO1-Asn240Gly rev) and primers used to create the mutant *jr*PPO2-Gly240Asn (*jr*PPO2-Gly240Asn fwd and *jr*PPO2-Gly240Asn rev). The mutation sites are indicated as small letters (*jr*PPO1-Asn240Gly fwd and *jr*PPO2-Gly240Asn fwd).

| **substrate** | **λ (nm)** | **ε_λ_ (M^-1^ cm^-1^)** |
| --- | --- | --- |
| tyramine (Figure S8) | 480^a^ | 3300^a^ |
| *L*-tyrosine (Figure S8) | 475^a^ | 3600^a^ |
| dopamine (Figure S8) | 480^a^ | 3300^a^ |
| *L*-DOPA (Figure S8) | 475^a^ | 3600^a^ |
| pyrogallol (Figure S4) | 446^a^ | 3000^a^ |
| protocatechuic acid (Figure S4) | 454^b^ | 213±3.0^b^ |
| gallic acid (Figure S4) | 346^b^ | 4140±220^b^ |
| ethyl gallate (Figure S4) | 449^b^ | 547±27^b^ |
| 4-hydroxyphenylacetic acid (Figure S4) | 395^a^ | 1601^a^ |
| coumaric acid (Figure S4) | 495^a^ | 2062^a^ |
| caffeic acid (Figure S4) | 495^a^ | 2062^a^ |
| quercetin* (Figure S5) | 522 ^b^ | 31825±1748^b^ |
| taxifolin* (Figure S5) | 516 ^b^ | 35325±1867^b^ |

**Table S2.** Extinction coefficients and wavelengths used to determine kinetic parameters. * indicates substrates that were measured after the addition of MBTH. ^a^ indicates previously reported values^7^ and ^b^ indicates values determined within the scope of this study.

| **substrate** | **µg*_jr_*_PPO1_/200 µl** | **µg*_jr_*_PPO2_/200 µl** |
| --- | --- | --- |
| tyramine (Figure S8) |  | 7.1 |
| *L*-tyrosine (Figure S8) |  | 33 |
| dopamine (Figure S8) |  | 0.71 |
| *L*-DOPA (Figure S8) |  | 0.043 |
| pyrogallol (Figure S4) | 1.5 | 0.17 |
| protocatechuic acid (Figure S4) | 25 | 2.4 |
| gallic acid (Figure S4) |  | 2.4 |
| ethyl gallate (Figure S4) | 100 | 1.5 |
| 4-hydroxyphenylacetic acid (Figure S4) | 6.4 | 4.9 |
| coumaric acid (Figure S4) | 8.5 | 47 |
| caffeic acid (Figure S4) | 6.4 | 1.1 |
| quercetin (Figure S5) | 19 | 4.7 |
| taxifolin (Figure S5) | 11 | 2.4 |

**Table S3.** Amount of enzyme (µg) used per 200 µl assay to determine kinetic parameters of *jr*PPO1 and *jr*PPO2.

| **substrate** | **enzyme** | **affinity (kcal/mol)**^a^ | **reasonable poses**^b^ |
| --- | --- | --- | --- |
| tyramine  (Figure S8) | *jr*PPO1 | 6.5 | 2/20 |
|  | *jr*PPO2 | 6.5 | 2/20 |
| *L*-tyrosine  (Figure S8) | *jr*PPO1 | 7.2 | 2/20 |
|  | *jr*PPO2 | 6.6 | 1/20 |
| dopamine  (Figure S8) | *jr*PPO1 | 5.6 | 1/20 |
|  | *jr*PPO2 | 6.4 | 4/20 |
| *L*-DOPA  (Figure S8) | *jr*PPO1 | 6.8 | 2/20 |
|  | *jr*PPO2 | 7.2 | 3/20 |
| pyrogallol  (Figure S4) | *jr*PPO1 | 6.0 | 2/20 |
|  | *jr*PPO2 | 5.3 | 2/20 |
| protocatechuic acid  (Figure S4) | *jr*PPO1 | 5.6 | 2/20 |
|  | *jr*PPO2 | 5.1 | 2/20 |
| gallic acid  (Figure S4) | *jr*PPO1 | * | * |
|  | *jr*PPO2 | 5.3 | 4/20 |
| ethyl gallate  (Figure S4) | *jr*PPO1 | 5.7 | 3/20 |
|  | *jr*PPO2 | 5.7 | 4/20 |
| 4-hydroxyphenylacetic acid (Figure S4) | *jr*PPO1 | 6.9 | 2/20 |
|  | *jr*PPO2 | 5.5 | 4/20 |
| coumaric acid  (Figure S4) | *jr*PPO1 | 6.9 | 1/20 |
|  | *jr*PPO2 | 7.2 | 2/20 |
| caffeic acid  (Figure S4) | *jr*PPO1 | 6.6 | 3/20 |
|  | *jr*PPO2 | 7.1 | 4/20 |
| quercetin  (Figure S5) | *jr*PPO1 | 8.2 | 3/20 |
|  | *jr*PPO2 | 7.5 | 5/20 |
| taxifolin  (Figure S5) | *jr*PPO1 | 8.3 | 1/20 |
|  | *jr*PPO2 | 7.9 | 4/20 |

**Table S4.** ^a^ represents the binding affinity calculated by AutoDock Vina^9^ for the most ‘reasonable’ binding pose. ^b^ indicates in how many of the 20 generated poses the phenolic hydroxy group of a substrate is oriented towards the di-copper center. * represents samples that were active but could not be measured due to an extensively reduced reactivity and an increased *K*_m_ value in combination with limited substrate solubility.

| **substrate** | **enzyme** | **affinity (kcal/mol)**^a^ | **reasonable poses**^b^ |
| --- | --- | --- | --- |
| tyramine  (Figure S8) | *jr*PPO1-Asn240Gly | 6.1 | 3/20 |
|  | *jr*PPO2-Gly240Asn | 6.3 | 1/20 |
| *L*-tyrosine  (Figure S8) | *jr*PPO1-Asn240Gly | 6.6 | 2/20 |
|  | *jr*PPO2-Gly240Asn | 6.7 | 1/20 |
| dopamine  (Figure S8) | *jr*PPO1-Asn240Gly | 5.6 | 3/20 |
|  | *jr*PPO2-Gly240Asn | 6.6 | 2/20 |
| *L*-DOPA  (Figure S8) | *jr*PPO1-Asn240Gly | 6.8 | 4/20 |
|  | *jr*PPO2-Gly240Asn | 7.1 | 3/20 |
| pyrogallol  (Figure S4) | *jr*PPO1-Asn240Gly | 5.1 | 3/20 |
|  | *jr*PPO2-Gly240Asn | 6.2 | 2/20 |
| protocatechuic acid  (Figure S4) | *jr*PPO1-Asn240Gly | 5.9 | 2/20 |
|  | *jr*PPO2-Gly240Asn | 5.7 | 1/20 |
| gallic acid  (Figure S4) | *jr*PPO1-Asn240Gly | 5.9 | 4/20 |
|  | *jr*PPO2-Gly240Asn | * | * |
| ethyl gallate  (Figure S4) | *jr*PPO1-Asn240Gly | 6.2 | 5/20 |
|  | *jr*PPO2-Gly240Asn | 6.6 | 3/20 |
| 4-hydroxyphenylacetic acid (Figure S4) | *jr*PPO1-Asn240Gly | 6.3 | 1/20 |
|  | *jr*PPO2-Gly240Asn | 7.0 | 1/20 |
| coumaric acid  (Figure S4) | *jr*PPO1-Asn240Gly | 6.7 | 2/20 |
|  | *jr*PPO2-Gly240Asn | 7.0 | 1/20 |
| caffeic acid  (Figure S4) | *jr*PPO1-Asn240Gly | 6.8 | 5/20 |
|  | *jr*PPO2-Gly240Asn | 7.1 | 3/20 |
| quercetin  (Figure S5) | *jr*PPO1-Asn240Gly | 8.8 | 4/20 |
|  | *jr*PPO2-Gly240Asn | 7.1 | 1/20 |
| taxifolin  (Figure S5) | *jr*PPO1-Asn240Gly | 8.5 | 7/20 |
|  | *jr*PPO2-Gly240Asn | 6.7 | 2/20 |

**Table S5.** ^a^ reports the binding affinity calculated by AutoDock Vina^9^ for the most ‘reasonable’ binding pose. ^b^ indicates in how many of the 20 generated poses the phenolic hydroxy group of a substrate is oriented towards the di-copper center. * represents samples that were active but could not be measured due to an extensively reduced reactivity and an increased *K*_m_ value, in combination with limited substrate solubility.

**3. Supplementary Figures**


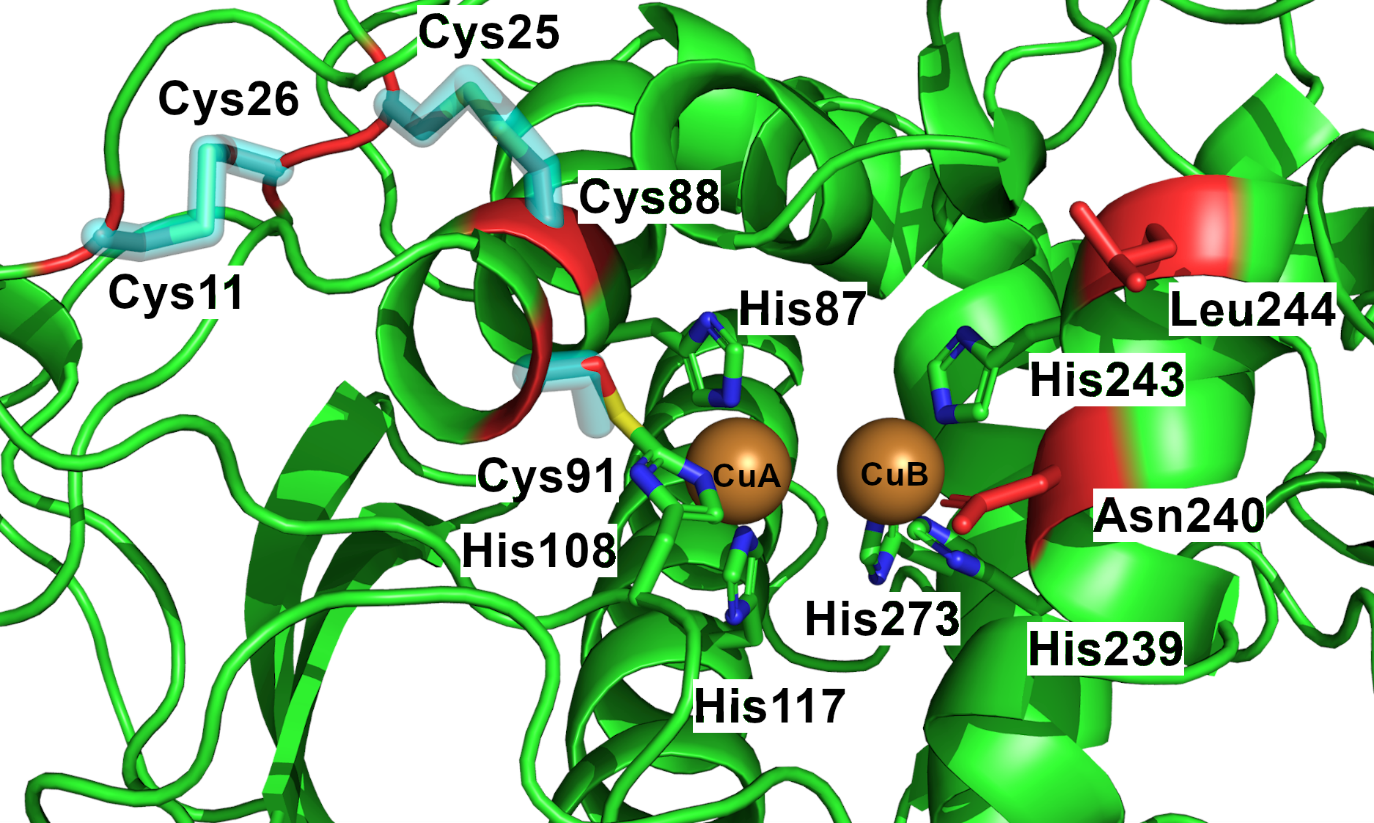


**Figure S1.** Active center of jrPPO1(PDB entry: 5CE9; green cartoon). The six conserved histidines coordinating CuA (His87, His108 and His117) and CuB (His239, His243 and His273) are shown as green sticks with blue nitrogen atoms (stick radius = 0.25 Å). The 1^st^ activity controller residue (Asn240), the 2^nd^ activity controller residue (Leu244) and the cysteines involved in the formation of the disulfide bonds (Cys11 - Cys26 and Cys25 - Cys88) and the thioether-bridge in jrPPO1 are shown as red sticks (stick radius = 0.25 Å) and the sulfur atoms of the cysteines are shown as yellow sticks. The cysteines involved in the formation of the disulfide bonds and the thioether bridge of jrPPO2 are shown (as calculated by the SWISS-MODEL server^10,11^) as cyan, transparent sticks (stick radius = 0.5 Å). The images were created using PyMOL 2.3^13^.


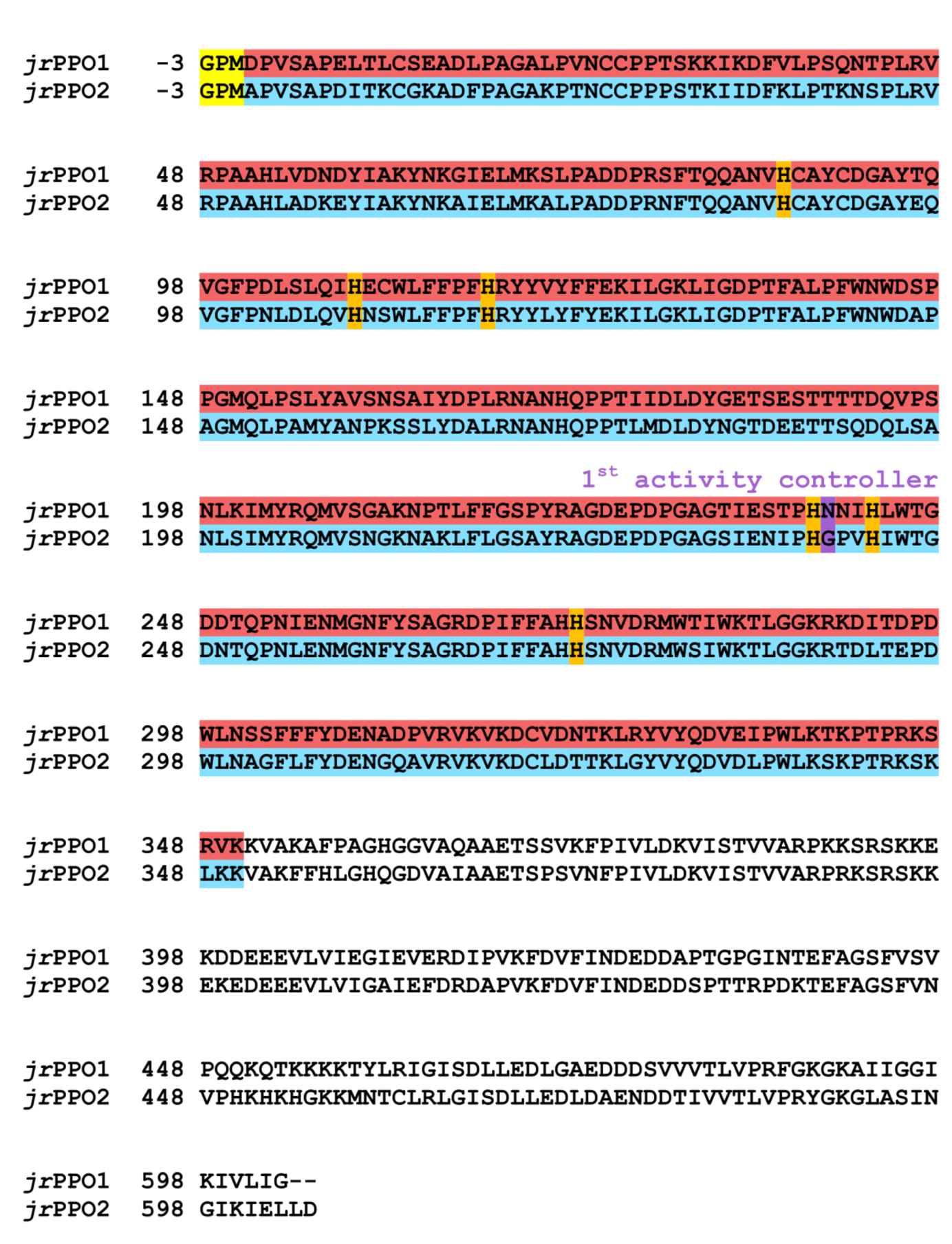
**Figure S2. Sequence alignment of *jr*PPO1 and *jr*PPO2.** The active domains of *jr*PPO1^1^ and *jr*PPO2 are colored in red and blue, respectively with the adjacent C-terminal domain left uncolored. The CuA and CuB coordinating histidines are colored in orange and the 1^st^ activity controller residues are highlighted in purple. The first three amino acids (yellow) result from the recombinant expression process.


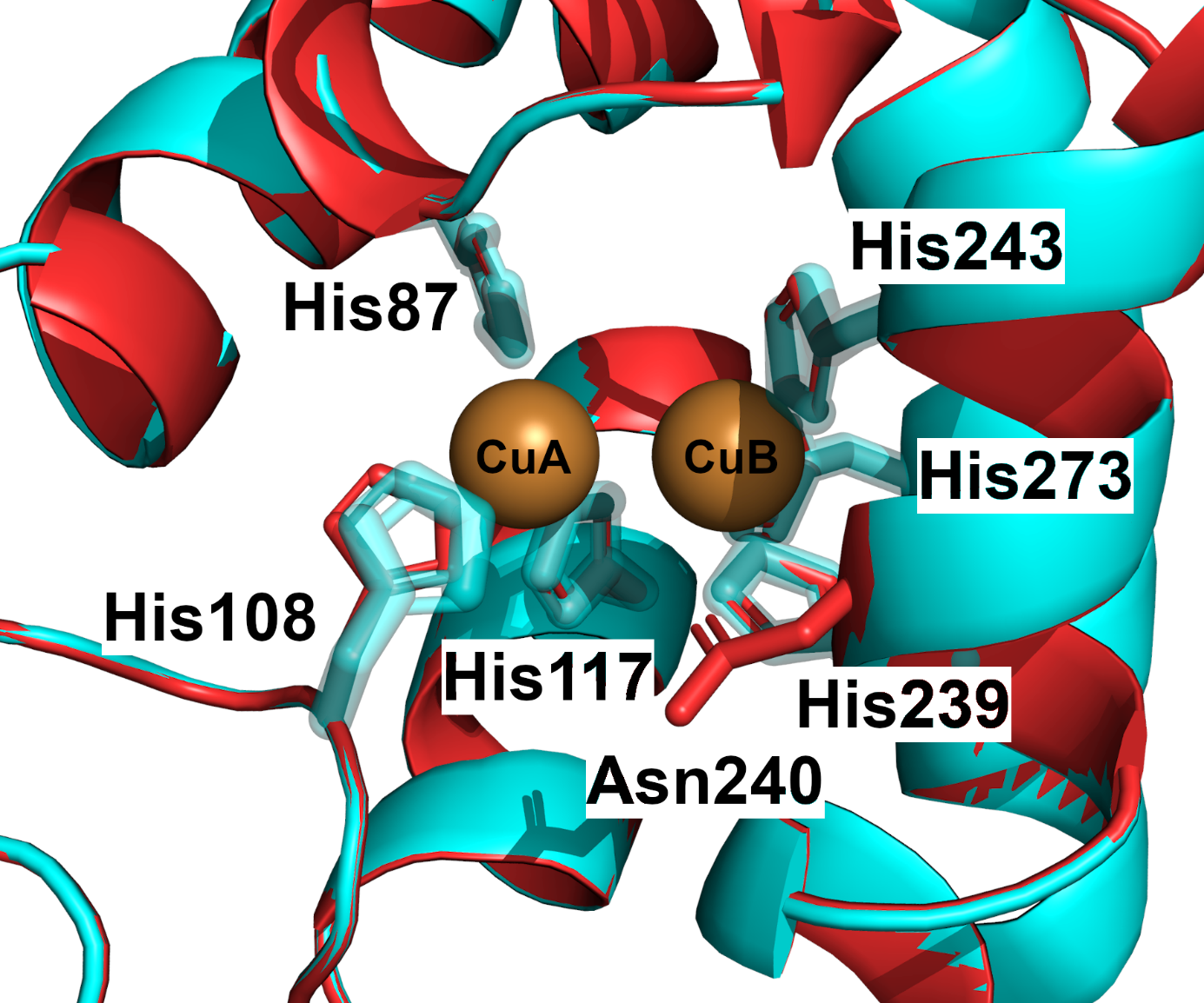


**Figure S3. Alignment of the crystal structure of *jr*PPO1**^12^ **(red) and the homology model of *jr*PPO2 (blue).** Both structures are depicted as cartoons, the copper ions are shown as brown spheres. The six His residues coordinating the two copper ions and the 1^st^ activity controller in position 240 are shown as sticks. *jr*PPO1: red sticks (stick radius = 0.25 Å); *jr*PPO2: transparent, blue sticks (stick radius = 0.4 Å). The images were created using PyMOL 2.3^13^.


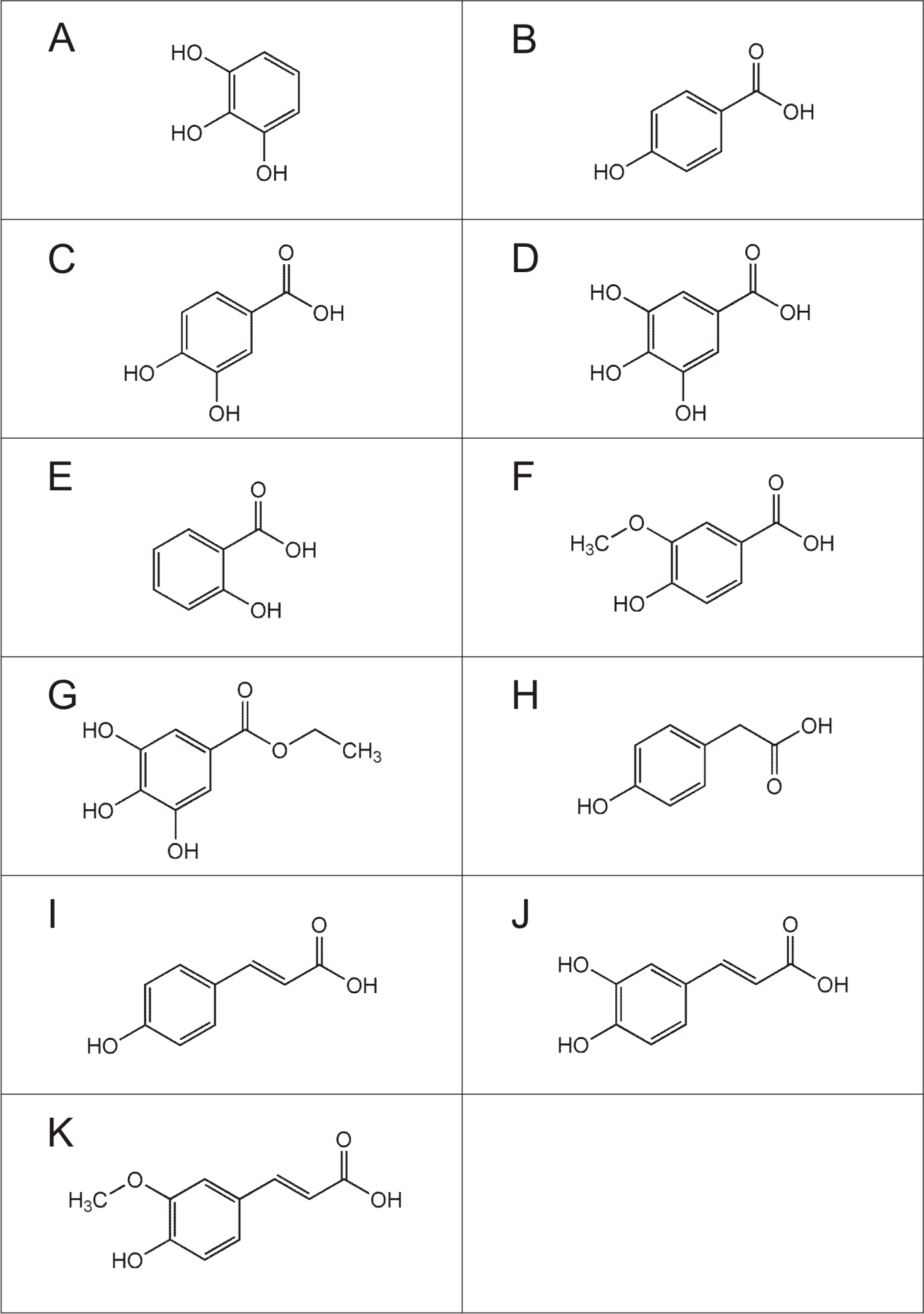


**Figure S4. Structures small-phenolic substrates naturally present in walnut.** A = pyrogallol, B = 4-hydroxybenzoic acid, C = protocatechuic acid, D= gallic acid, E = salicylic acid, F = vanillic acid, G = ethyl gallate, H = 4-hydroxyphenylacetic acid, I = coumaric acid, J = caffeic acid, K = ferulic acid.


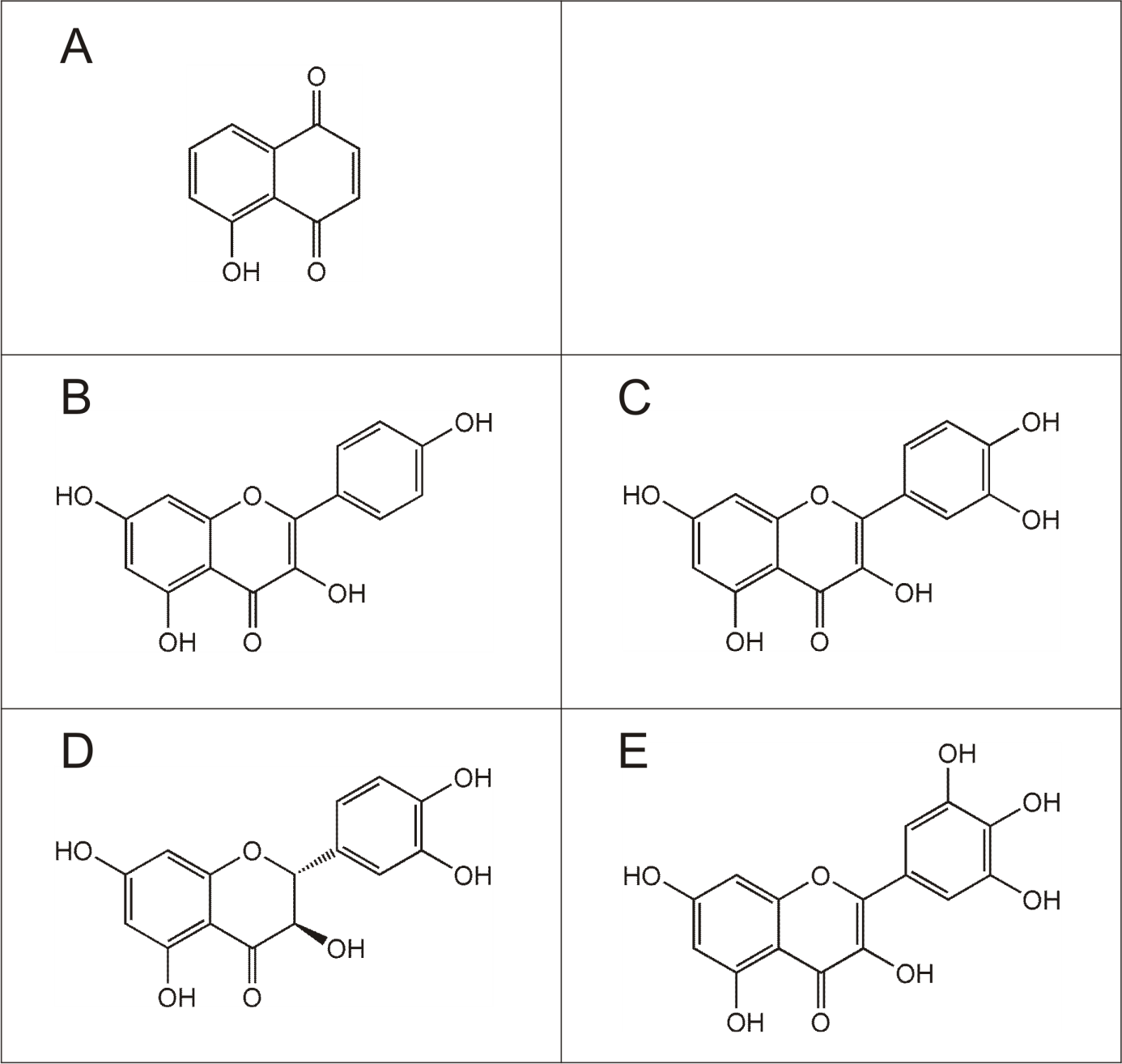


**Figure S5. Structures of naphthoquinone and flavonoid substrates naturally present in walnut.** A = juglone, B = kaempferol, C = quercetin, D = taxifolin, E = myricetin.


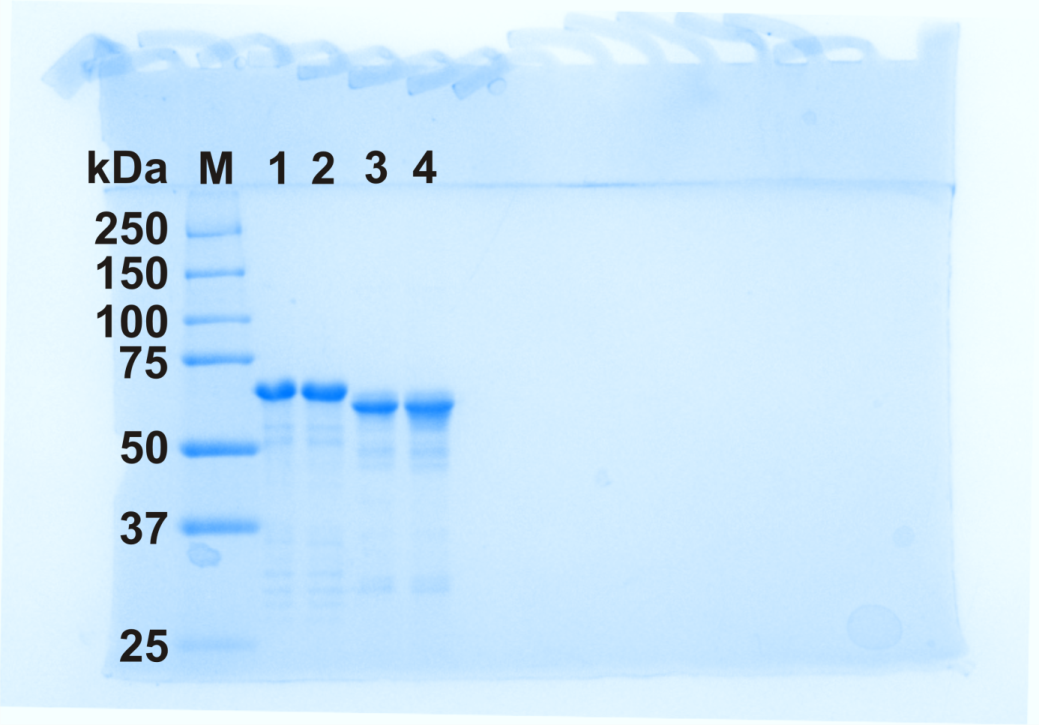


**Figure S6. Full-length SDS PAGE of recombinantly expressed purified, latent enzymes.** M = Marker, 1 = *jr*PPO2, 2 = *jr*PPO2-Gly240Asn, 3 = *jr*PPO1, 4 = *jr*PPO1-Asn240Gly. The gel was satined using Coomassie Brilliant Blue G250 (Fluka, Buchs, Switzerland) and the image was recorded on a Bio-Rad Unviersa Hood II using the Image Lab v. 5.2.1 software.


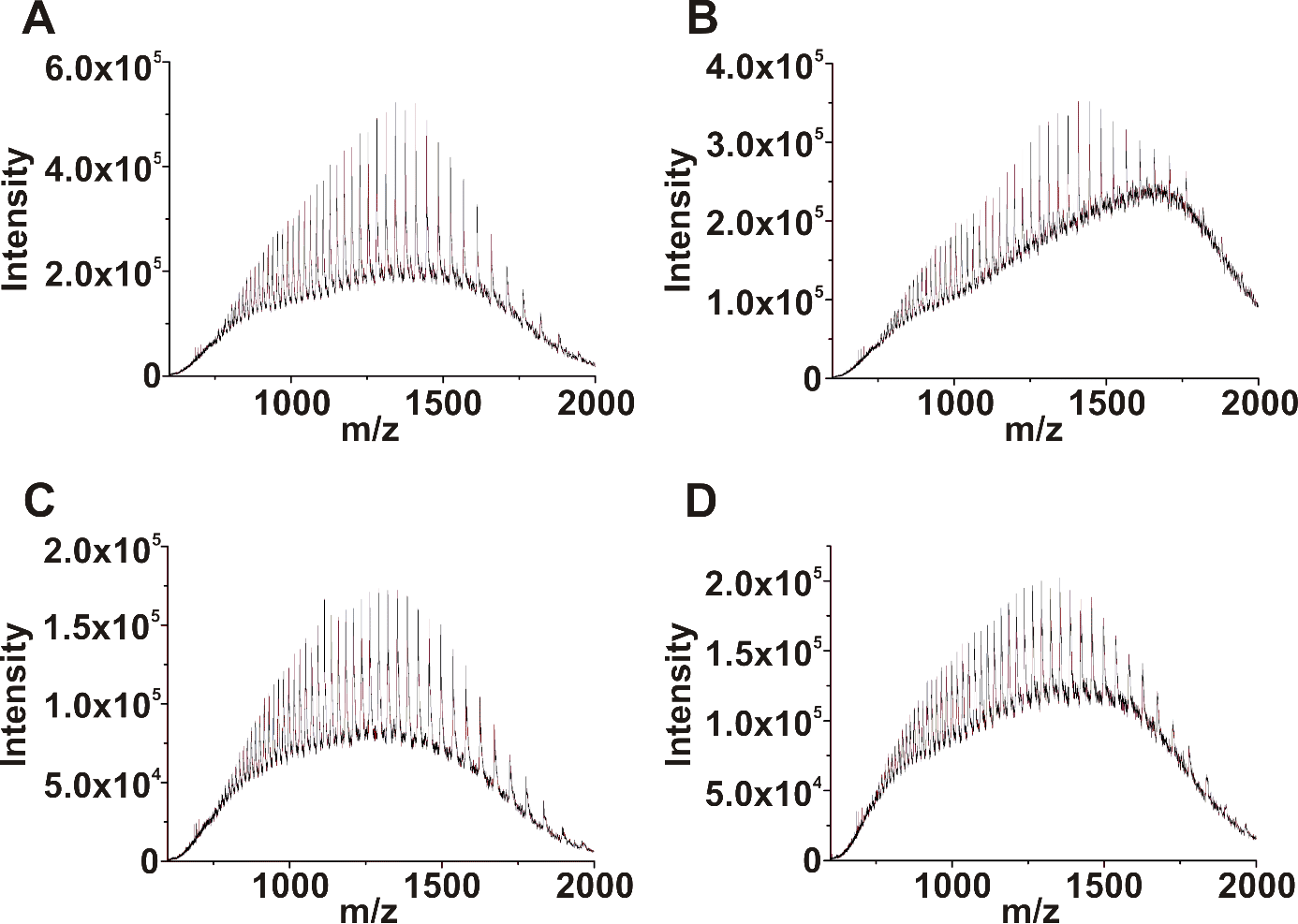


**Figure S7. Positive mode ESI-LTQ-Orbitrap Velos mass spectra.** A = *jr*PPO1, B = *jr*PPO1-Asn240Gly, C = *jr*PPO2, D = *jr*PPO2-Gly240Asn. The calculated and measured masses of the proteins are listed in Table 1 of the main manuscript.


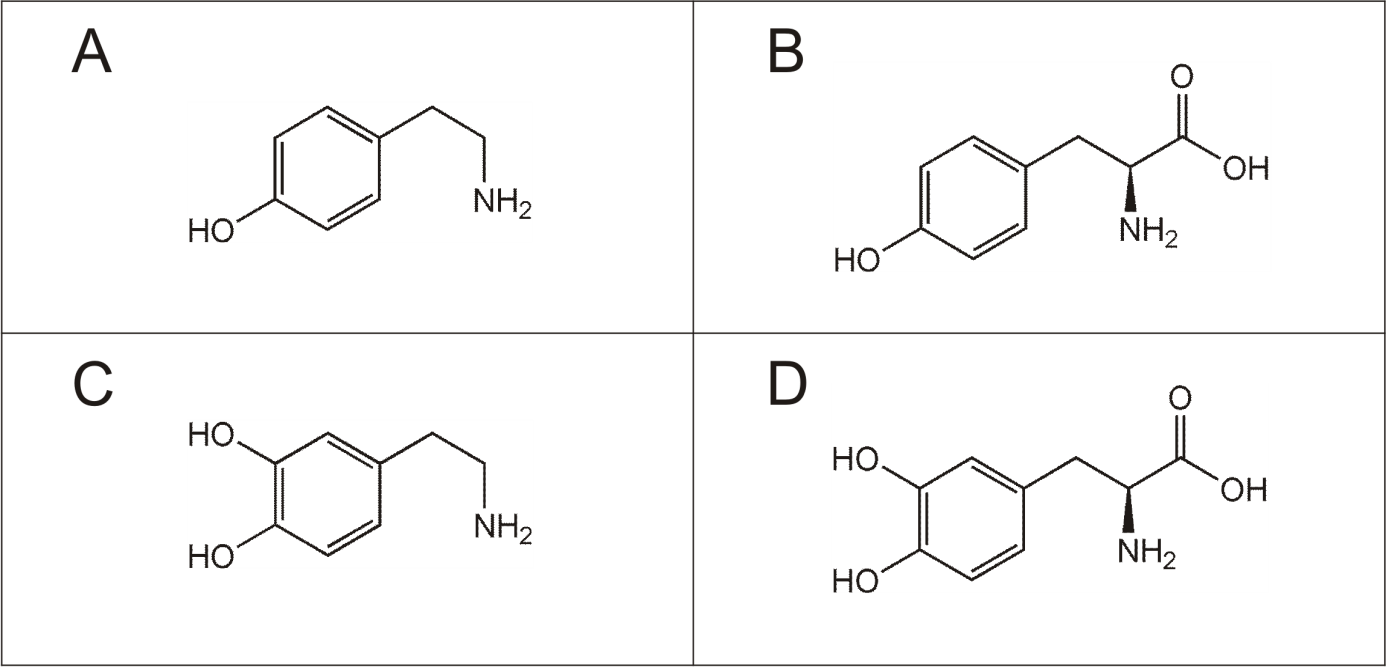


**Figure S8. Structures of standard substrates.** A = tyramine, B = *L*-tyrosine, C = dopamine, D = *L*-DOPA.


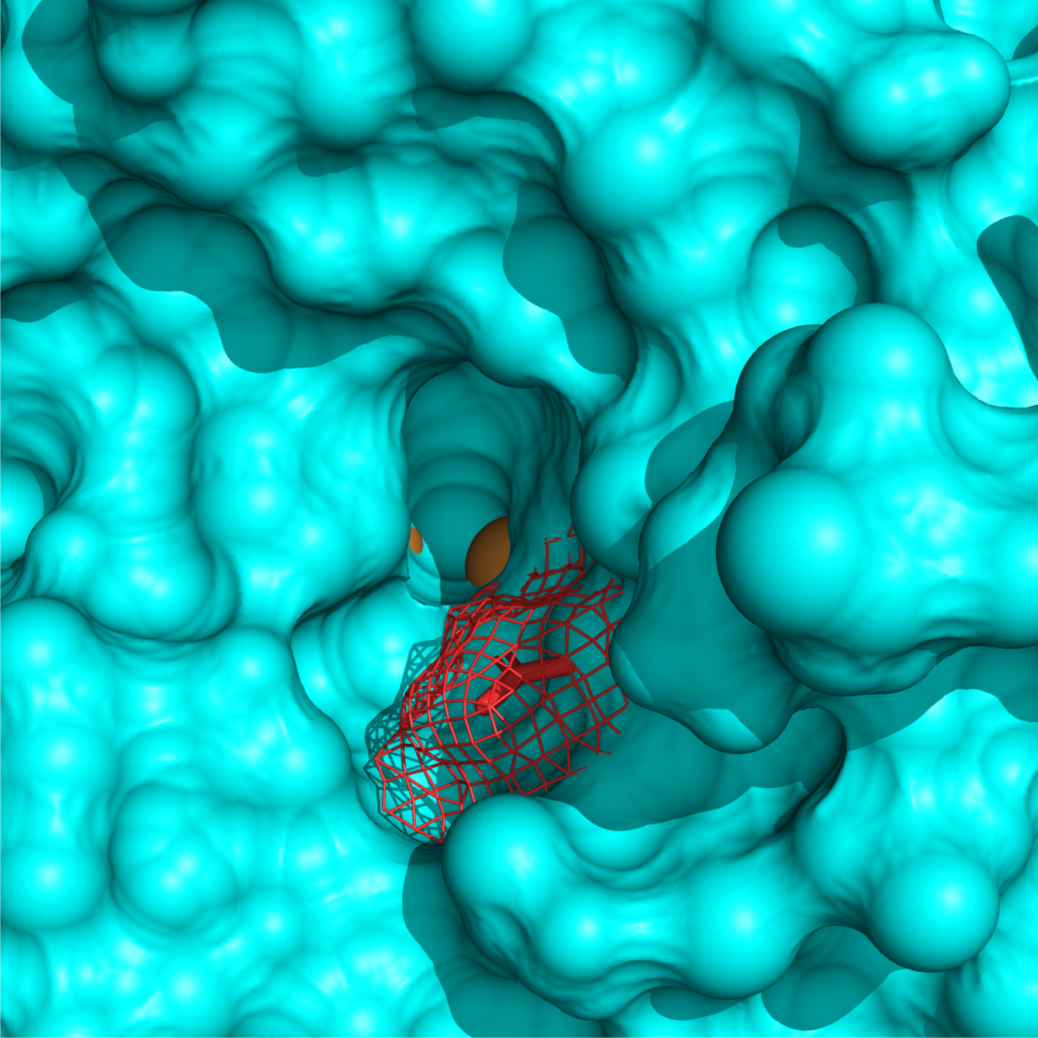


**Figure S9. Active center of *jr*PPO2 (blue surface) and the 1^st^ activity controller residue (Asn240) of *jr*PPO1 (red mesh and red sticks).** The red mesh illustrates the space occupied by Asn240 in *jr*PPO1, which, in contrast, is openly accessible in *jr*PPO2. The two copper ions are partially visible as brown spheres. The images were created using PyMOL 2.3^13^.


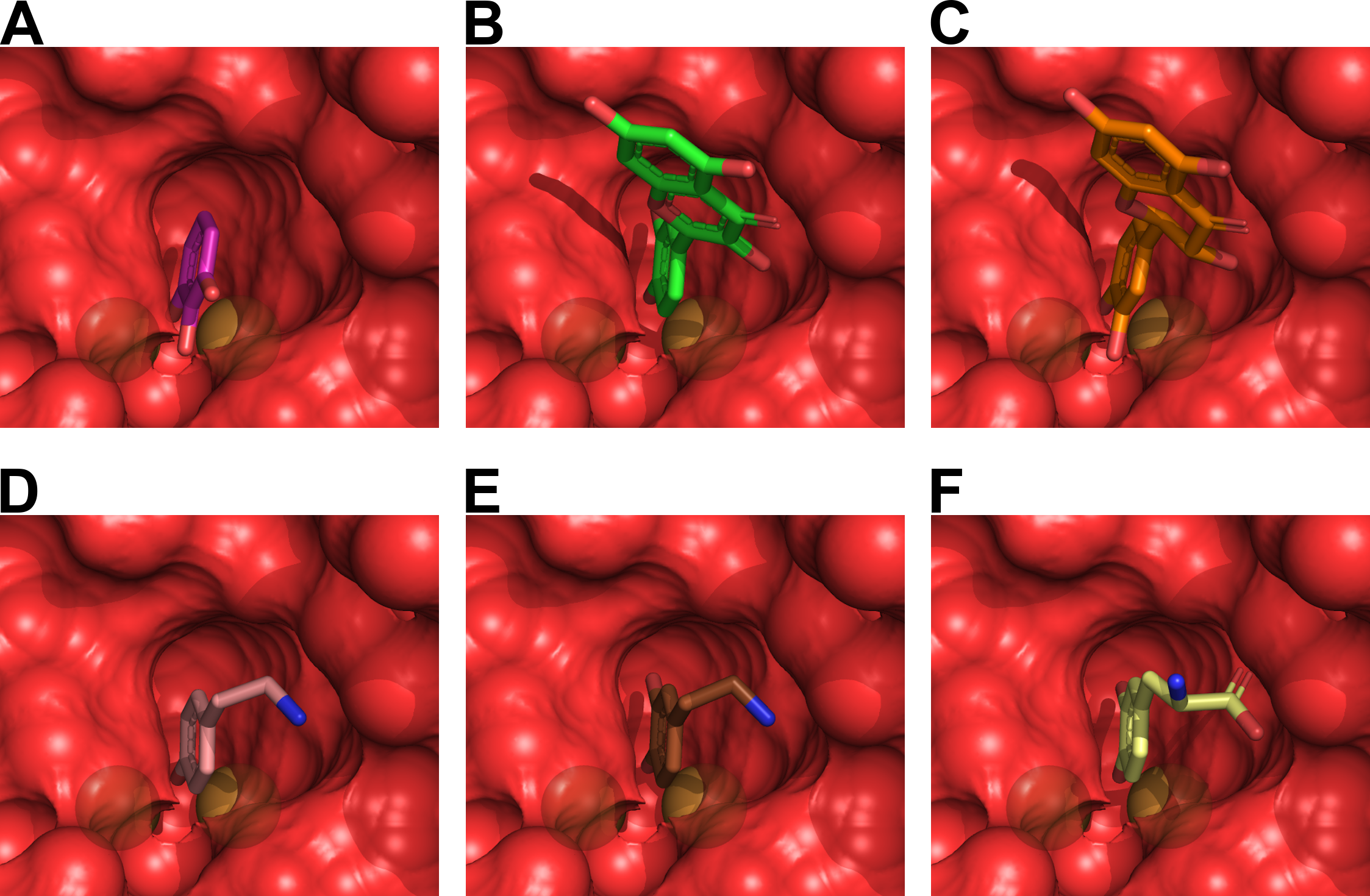


**Figure S10. Docking poses calculated for *jr*PPO1.** The transparency was set to 0.3. The copper ions are displayed as brown spheres. A = pyrogallol, B = quercetin, C = taxifolin, D = tyramine, E = dopamine, F = *L*-DOPA (Figures S4, S5 and S8). The images were created using PyMOL 2.3^13^ and edited using GIMP 2.10.18 (https://www.gimp.org).


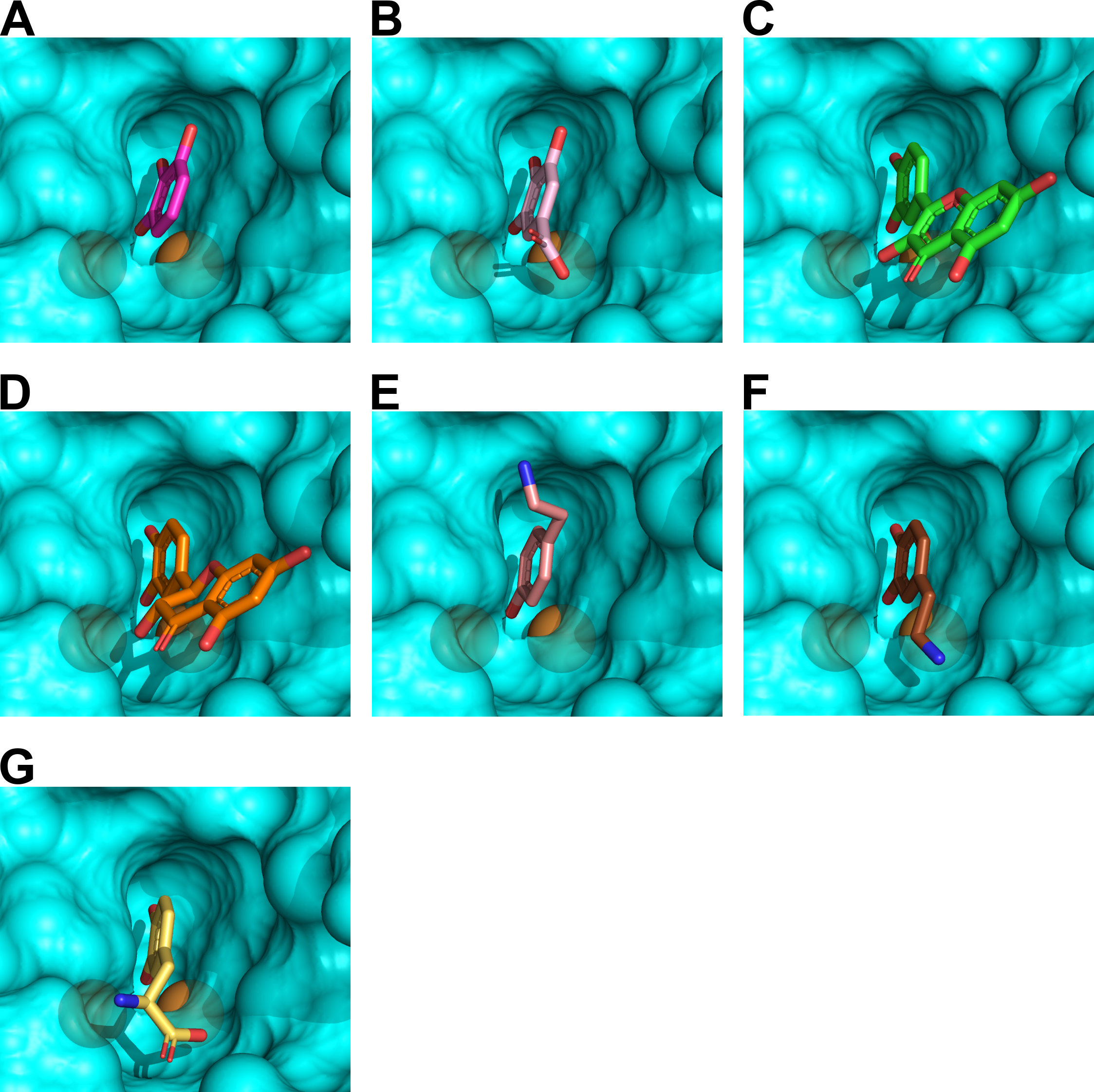


**Figure S11. Docking poses calculated for *jr*PPO2.** The transparency was set to 0.3. The copper ions are displayed as brown spheres. A = pyrogallol, B = gallic acid, C = quercetin, D = taxifolin, E = tyramine, F = dopamine, G = *L*-DOPA (Figures S4, S5 and S8). The images were created using PyMOL 2.3^13^ and edited using GIMP 2.10.18 (https://www.gimp.org).


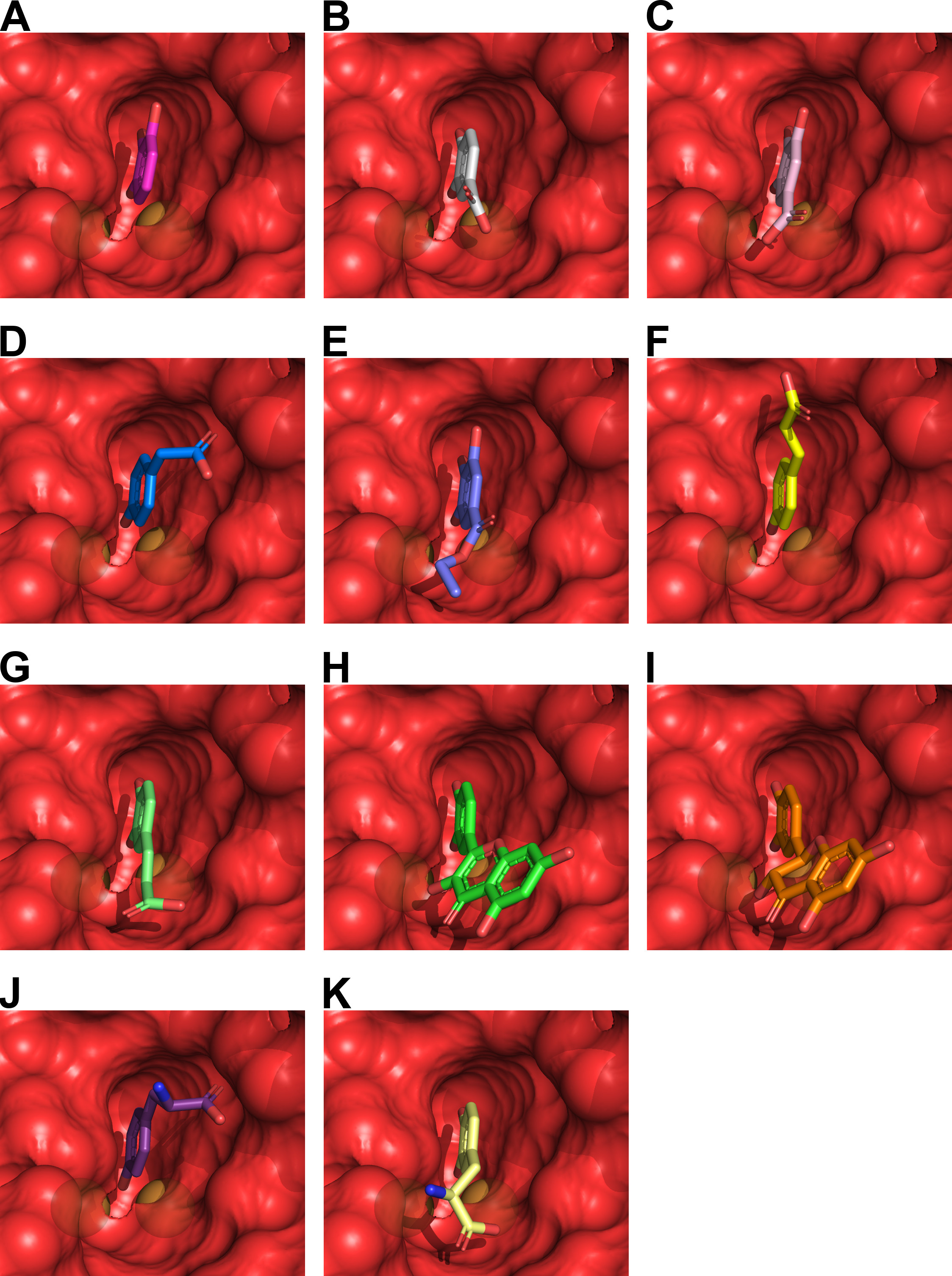


**Figure S12. Docking poses calculated for *jr*PPO1-Asn240Gly.** The transparency was set to 0.3. The copper ions are displayed as brown spheres. A = pyrogallol, B = protocatechuic acid, C = gallic acid, D = 4-hydroxyphenylacetic acid, E = ethyl gallate, F = coumaric acid, G = caffeic acid, H = quercetin, I = taxifolin, J = tyrosine, K = *L*-DOPA (Figures S4, S5 and S8). The images were created using PyMOL 2.3^13^ and edited using GIMP 2.10.18 (https://www.gimp.org).


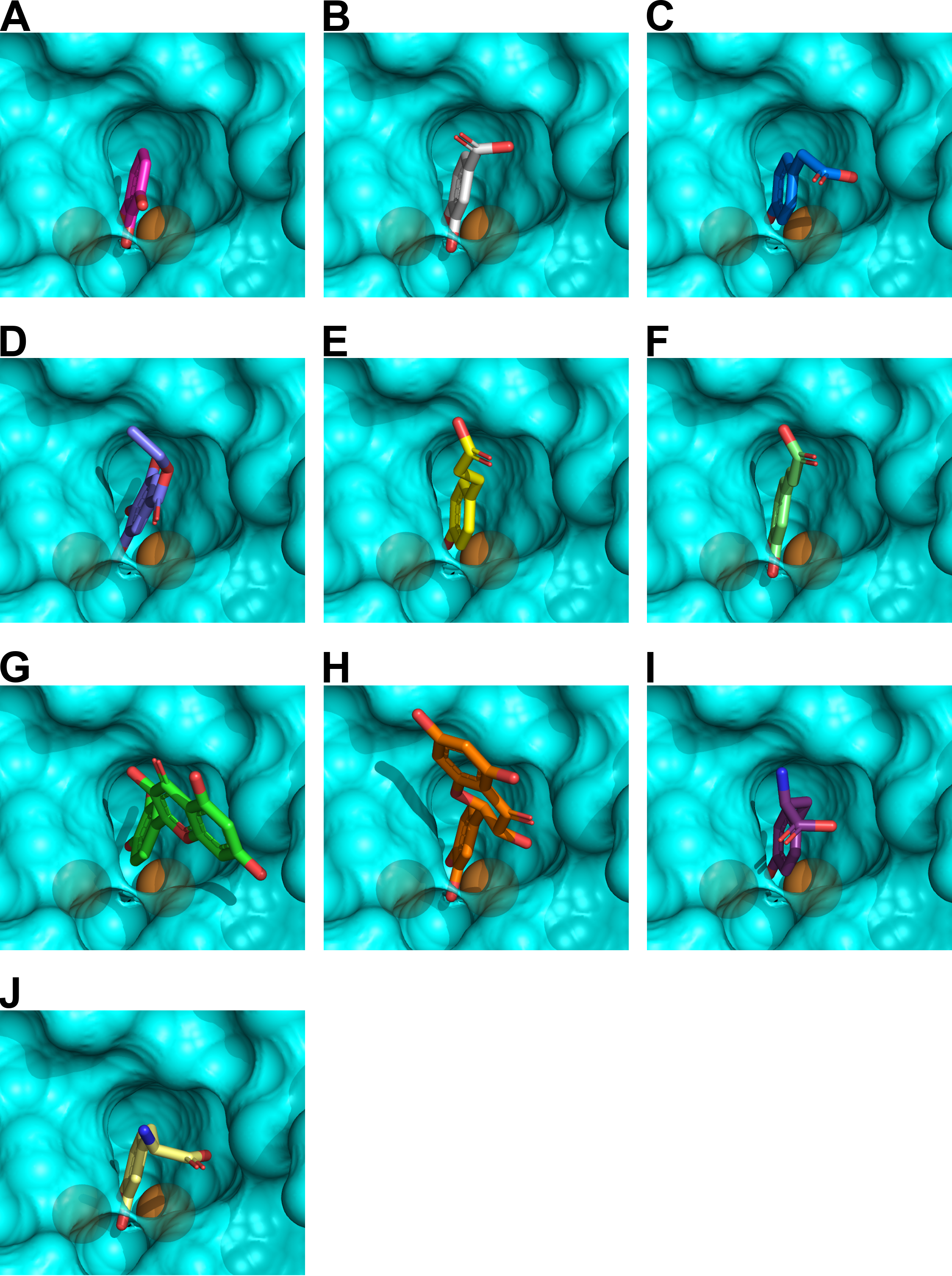


**Figure S13. Docking poses calculated for *jr*PPO2-Gly240Asn.** The transparency was set to 0.3. The copper ions are displayed as brown spheres. A = pyrogallol, B = protocatechuic acid, C = 4-hydroxyphenylacetic acid, D = ethyl gallate, E = coumaric acid, F = caffeic acid, G = quercetin, H = taxifolin, I = tyrosine, J = *L*-DOPA (Figures S4, S5 and S8). The images were created using PyMOL 2.3^13^ and edited using GIMP 2.10.18 (https://www.gimp.org).


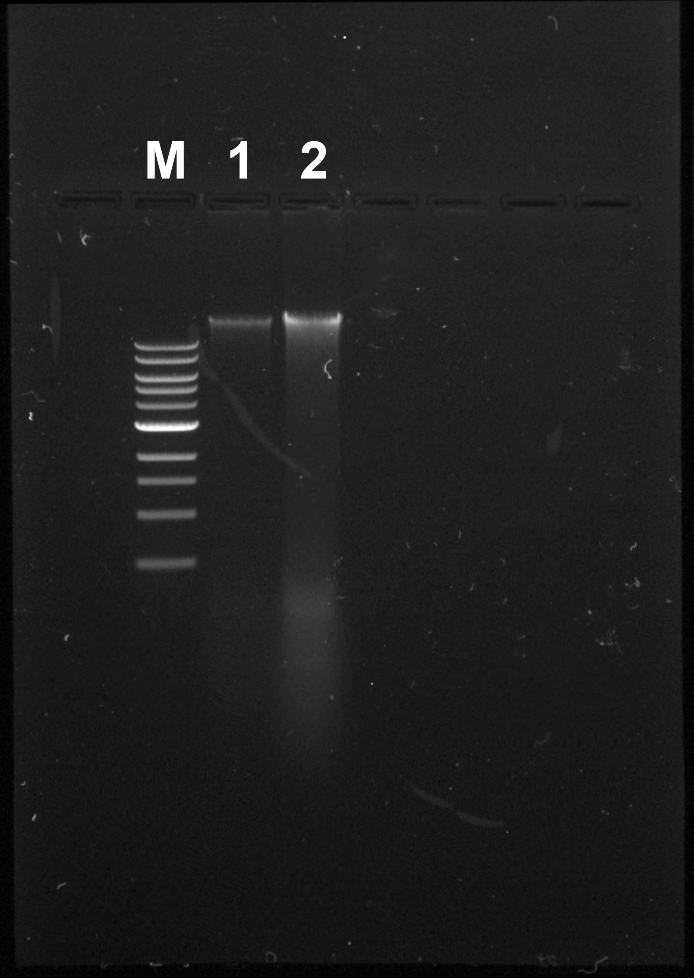


**Figure S14.** **Full length agarose gel (0.6 %) of gDNA extracted from walnut leaves.** M = marker (NEB, 1 kb DNA ladder. The bands correspond to a length of 10, 8, 6, 5, 4, 3, 2, 1.5, 1, 0.5 kilobasepairs), 1 = 4 µl gDNA extract, 2 = 20 µl gDNA extract. The gel was run at 5 V/cm. The gel was stained using SYBR Safe DNA Gel Stain (Thermo Fisher Scientific, Bremen, Germany) and the image was recorded on a Bio-Rad Unviersa Hood II using the Image Lab v. 5.2.1 software.


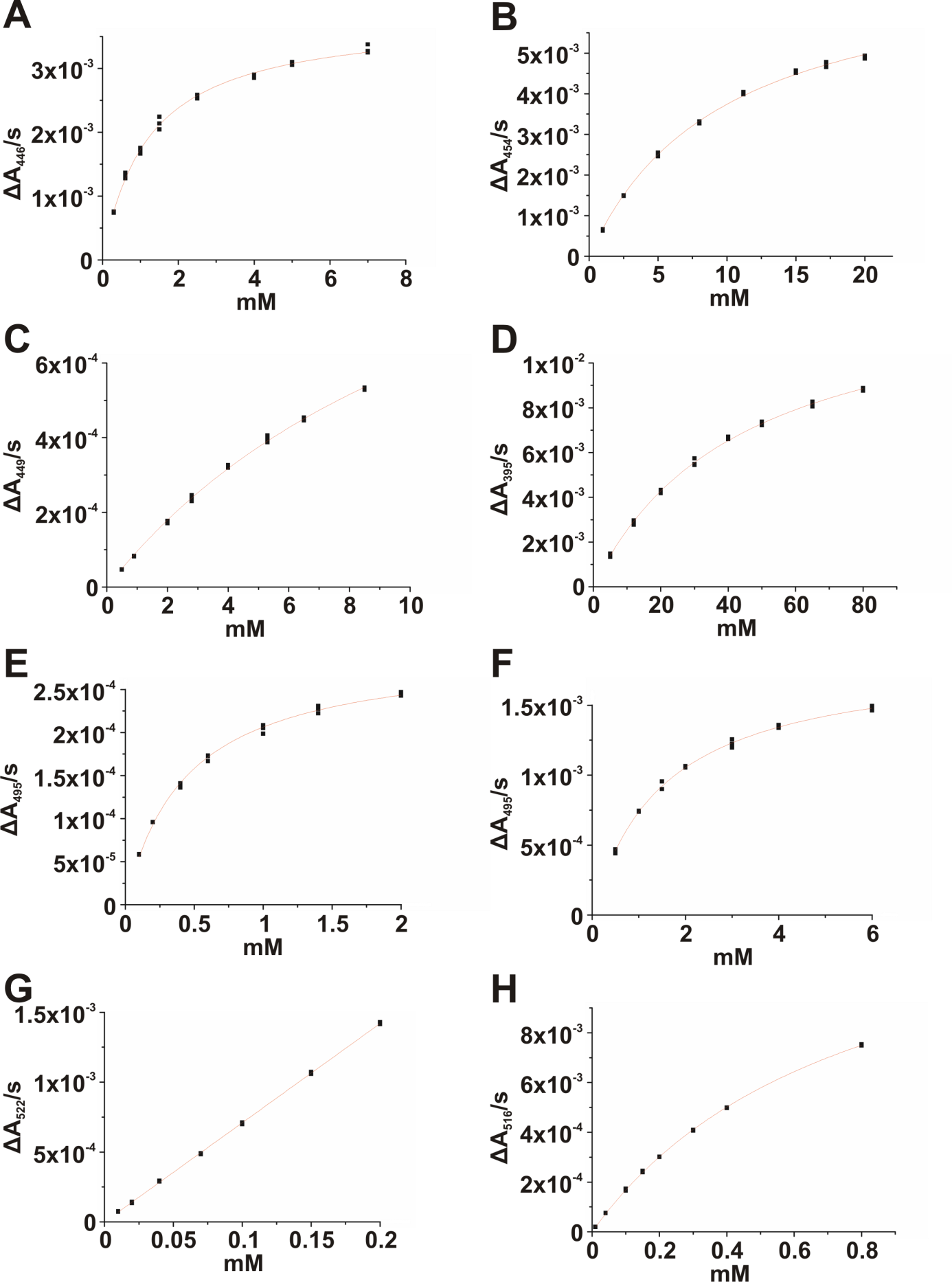


**Figure S15. Non-liner curve fitting of data points measured for *jr*PPO1 and the natural substrates during kinetic assays.** Triplets were fitted using the Michaelis-Menten equation and the least-squares method built in the OriginPro 8 software. A = pyrogallol, B = protocatechuic acid, C = ethyl gallate, D = 4-hydroxyphenylacetic acid, E = coumaric acid, F = caffeic acid, G = quercetin, H = taxifolin.


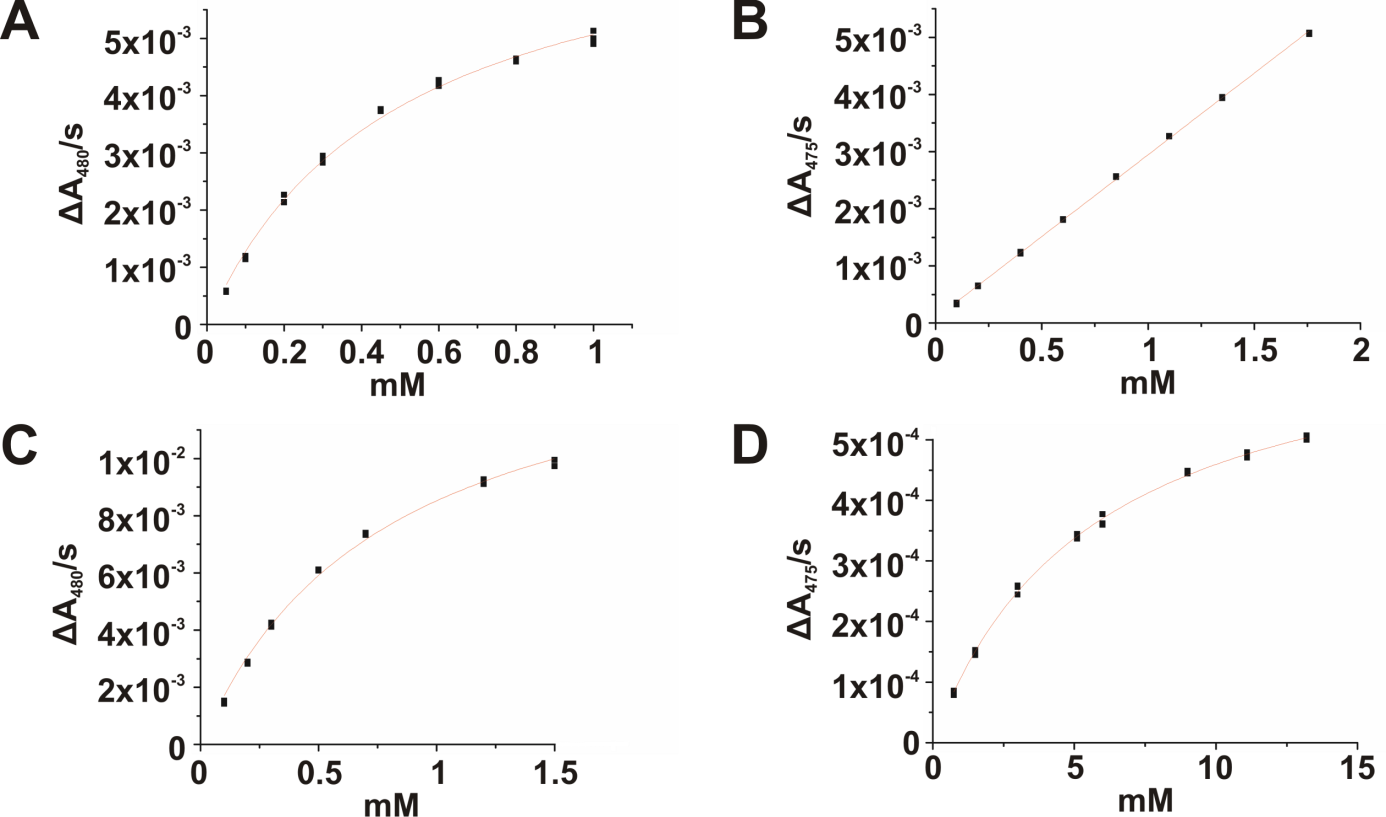


**Figure S16. Non-liner curve fitting of data points measured for *jr*PPO2 and the standard substrates during kinetic assays.** Triplets were fitted using the Michaelis-Menten equation and the least-squares method built in the OriginPro 8 software. A = tyramine, B = *L*-tyrosine, C = dopamine, D = *L*-DOPA.


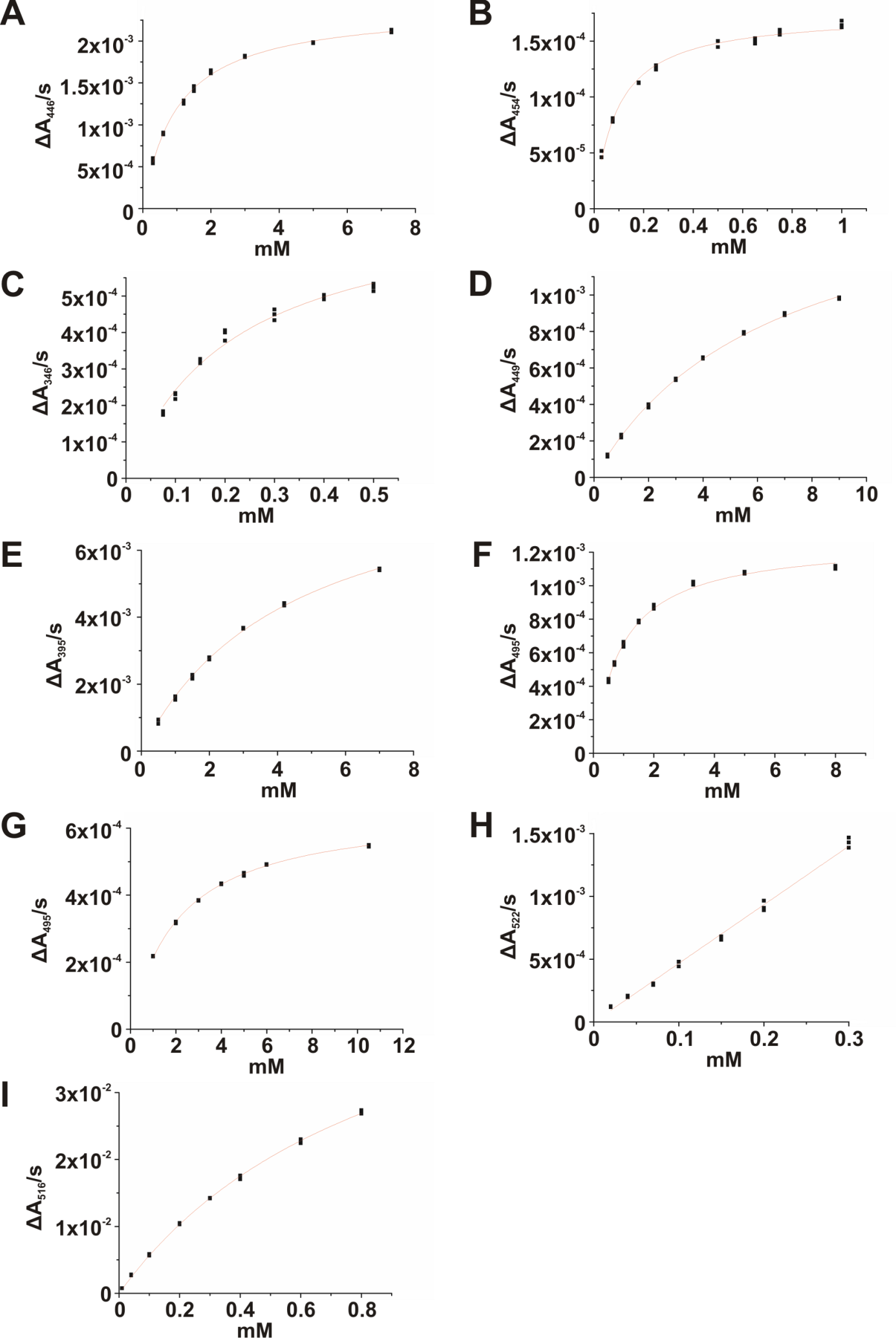


**Figure S17. Non-liner curve fitting of data points measured for *jr*PPO2 and natural substrates during kinetic assays.** Triplets were fitted using the Michaelis-Menten equation and the least-squares method built in the OriginPro 8 software. A = pyrogallol, B = protocatechuic acid, C = gallic acid, D = ethyl gallate, E = 4-hydroxyphenylacetic acid, F = coumaric acid, G = caffeic acid, H = quercetin, I = taxifolin.


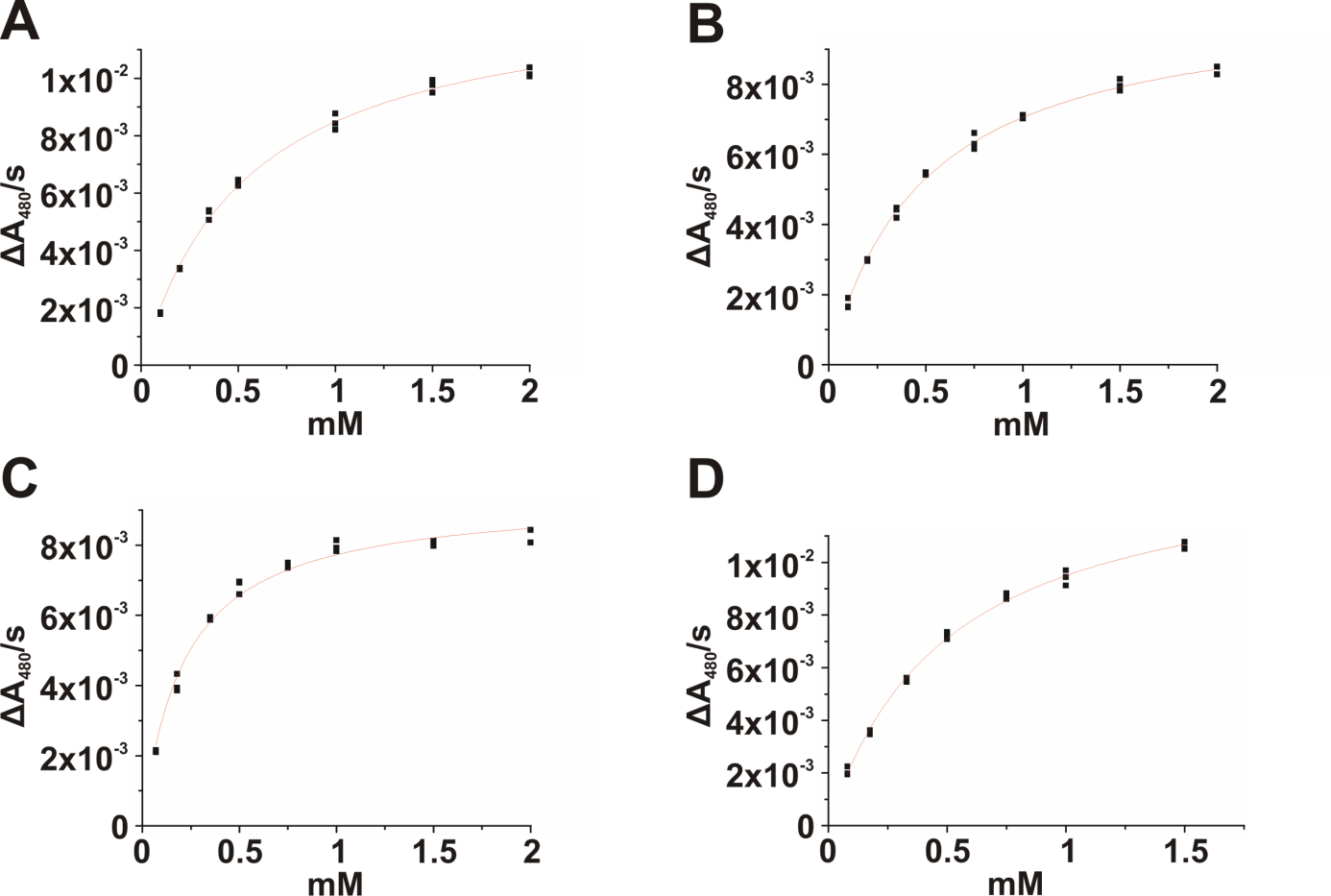


**Figure S18. Non-liner curve fitting of data points measured for *jr*PPO1-Asn240Gly (A and B) and *jr*PPO2-Gly240Asn (C and D).** Triplets were fitted using the Michaelis-Menten equation and the least-squares method built in the OriginPro 8 software. A = tyramine, B = dopamine, C = tyramine, D = dopamine.


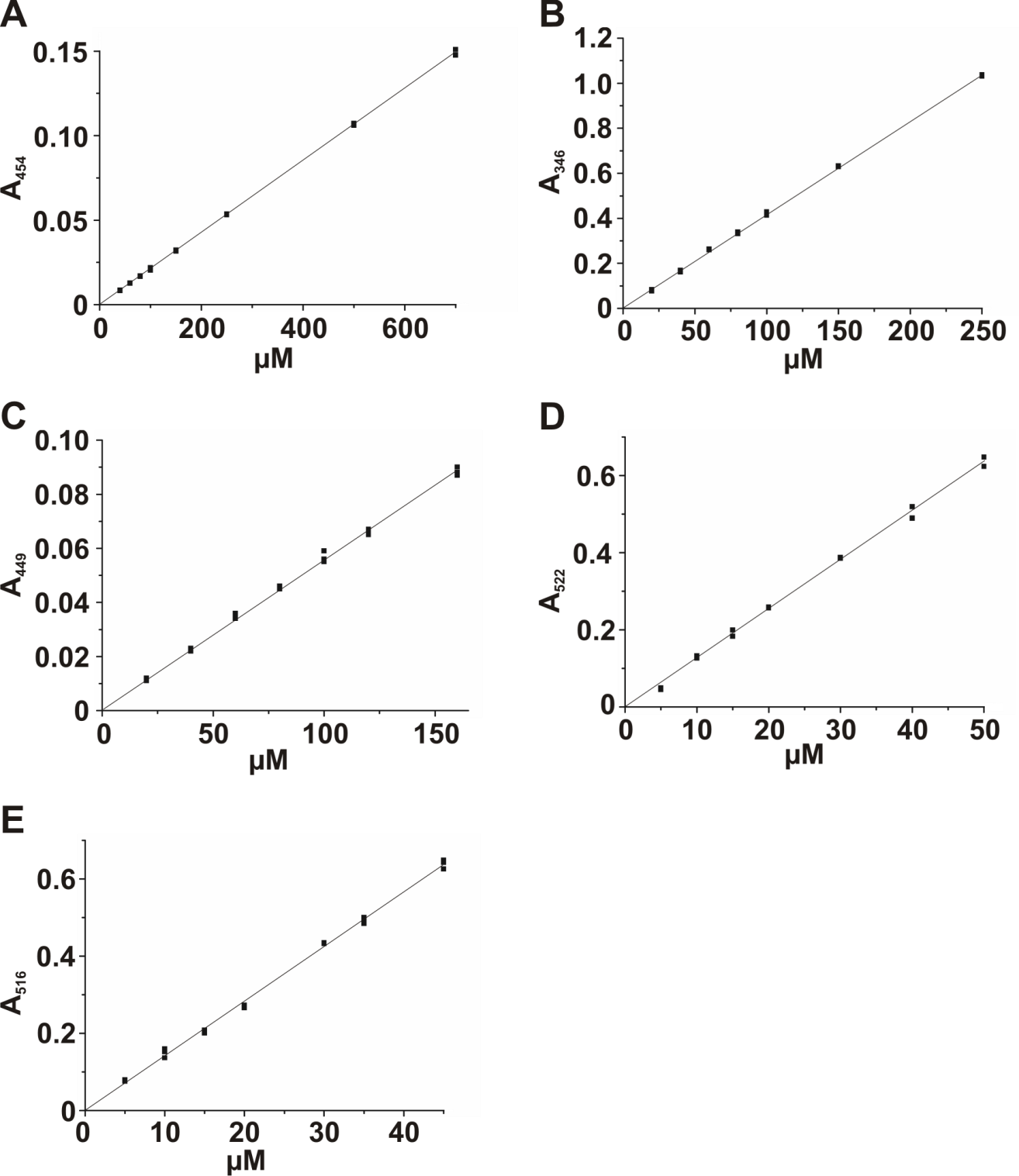


**Figure S19.** Regression lines used to calculate the molar extinction coefficients were calculated by linear curve fitting using the least-squares method built in the OriginPro 8 software. A = protocatechuic acid, B = gallic acid, C = ethyl gallate, D = quercetin, E = taxifolin.

**4. References**

1. Panis, F., Kampatsikas, I., Bijelic, A. & Rompel, A. Conversion of walnut tyrosinase into a catechol oxidase by site directed mutagenesis. *Sci. Rep.* **10**, 10659, doi:10.1038/s41598-020-57671-x (2020).

2. Pretzler, M., Bijelic, A. & Rompel, A. Heterologous expression and characterization of functional mushroom tyrosinase (*Ab*PPO4). *Sci. Rep.* **7**, 1810, doi:10.1038/s41598-017-01813-1 (2017).

3. Gasteiger, E. *et al*. The Proteomics Protocols Handbook. 571–608, doi:10.1385/1592598900 (2005).

4. Escobar, M. A., Shilling, A., Higgins, P., Uratsu, S. L. & Dandekar, A. M. Characterization of Polyphenol Oxidase from Walnut. *J. Am. Soc. Hortic. Sci.* **133**, 852–858, doi:10.21273/JASHS.133.6.852 (2008).

5. [Martínez-García](https://www.ncbi.nlm.nih.gov/pubmed/?term=Mart%C3%ADnez-Garc%C3%ADa%20PJ%5BAuthor%5D&cauthor=true&cauthor_uid=27145194), P. J. *et al.* The walnut (*Juglans regia*) genome sequence reveals diversity in genes coding for the biosynthesis of non-structural polyphenols. [*Plant J.*](https://www.ncbi.nlm.nih.gov/pubmed/27145194) **87**, 507–532, doi:10.1111/tpj.13207 (2016).

6. Hanna, P. M., Tamilarasan, R. & McMillin, D. R. Cu(I) analysis of blue copper proteins. *Biochem. J.* **256**, 1001–1004, doi:10.1042/bj2561001 (1988).

7. Muñoz, J. L. *et al*. Calculating molar absorptivities for quinones: Application to the measurement of tyrosinase activity. *Anal. Biochem.* **351**, 128–138, doi:10.1016/j.ab.2006.01.011 (2006).

8. Winder, A. J. & Harris, H. New assays for the tyrosine hydroxylase and dopa oxidase activities of tyrosinase. *Eur. J. Biochem*. **198**, 317–326, doi:10.1111/j.1432-1033.1991.tb16018.x (1991).

9. Trott, O. & Olson, A. Autodock vina: improving the speed and accuracy of docking. *J. Comput. Chem.* **31**, 455–461, doi:10.1002/jcc.21334 (2010).

10. Arnold, K., Bordoli, L., Kopp, J. & Schwede, T. The SWISS-MODEL workspace: a web-based environment for protein structure homology modelling. *Bioinformatics* **22**, 195–201, doi:10.1093/bioinformatics/bti770 (2006).

11. Bordoli, L. *et al*. Protein structure homology modeling using SWISS-MODEL workspace. *Nat. Protoc.* **4**, 1–13, doi:10.1038/ nprot.2008.197 (2008).

12. Bijelic, A., Pretzler, M., Molitor, C., Zekiri, F. & Rompel, A. The Structure of a Plant Tyrosinase from Walnut Leaves Reveals the Importance of “Substrate-Guiding Residues” for Enzymatic Specificity. *Angew. Chemie Int. Ed*. **54**, 14677–14680, doi:10.1002/anie.201506994 (2015) and Kristallstruktur einer pflanzlichen Tyrosinase aus Walnussblätter: Die Bedeutung "substratlenkender Aminosäurenreste" für die Enzymspezifität. *Angew. Chem.* **127**, 14889–14893, doi:10.1002/ ange.201506994 (2015).

13. The PyMOL Molecular Graphics System, Version 2.0 Schrödinger, LLC.
